# Supplementary material for: Trifunctional sphingomyelin derivatives enable nanoscale resolution of sphingomyelin turnover in physiological and infection processes via expansion microscopy
Source: Nat Commun. 2024 Aug 28;15:7456. doi: 10.1038/s41467-024-51874-w (PMC11358447; doi:10.1038/s41467-024-51874-w)
Supplement: Supplementary file 1 — Supplementary Information [file 41467_2024_51874_MOESM1_ESM.pdf]

Supplementary Information

Trifunctional sphingomyelin derivatives enable nanoscale resolution of sphingomyelin turnover in physiological and infection processes via expansion microscopy

Marcel Rühling[1], Louise Kersting[2*], Fabienne Wagner[1], Fabian Schumacher[3], Dominik Wigger[3], Dominic A. Helmerich[4], Tom Pfeuffer[2], Robin Elflein[2], Christian Kappe[5], Markus Sauer[4], Christoph Arenz[5], Burkhard Kleuser[3], Thomas Rudel[1], Martin Fraunholz[1§], and Jürgen Seibel[2§+]*

[1] Chair of Microbiology, Julius-Maximilians-University Würzburg, Theodor-Boveri-Weg, 97074 Würzburg, Germany

[2] Institute of Organic Chemistry, Julius-Maximilians-University Würzburg, Am Hubland, 97074 Würzburg, Germany

[3] Institute of Pharmacy, Freie Universität Berlin, Königin-Luise-Straße 2+4, 14195 Berlin, Germany

[4]Chair of Biotechnology & Biophysics, Biocenter, Julius-Maximilians-University Würzburg, Am Hubland, 97074 Würzburg, Germany

[5]Institute of Chemistry, Humboldt Universität zu Berlin, Brook-Taylor-Str 2, 12489 Berlin, Germany

*These authors contributed equally.

[§]These authors jointly supervised this work.

[+]Corresponding author: seibel@chemie.uni-wuerzburg.de

Table of Contents

Abbreviations.......... 3

Supplementary Methods.......... 3

General Information for Chemical Synthesis.......... 3

Chemical Synthesis.......... 4

LC-MS/MS Analysis.......... 18

Supplementary Figures.......... 21

NMR Spectra.......... 27

Supplementary Tables.......... 41

Supplementary References.......... 41

Abbreviations

anh: anhydrous; ar: aromatic; Boc: *tert*-butyloxycarbonyl; calcd: calculated; CH: cyclohexane; DCM: dichloromethane, DIPEA: *N*,*N*-diisopropylethylamine; DMF: *N*,*N*-dimethylformamide, EtOAc: ethyl acetate; equiv.: equivalents; iPrOH: iso-propanol; MeCN: acetonitrile; MeOH: methanol; MOM: methoxymethyl; TBAF: tetra-*n*-butylammonium fluoride; TBS: *tert*-butyldimethylsilyl; TFA: trifluoroacetic acid; THF: tetrahydrofuran; TLC: thin layer chromatography.

Supplementary Methods

General Information for Chemical Synthesis

Air- and moisture-sensitive reactions were performed under nitrogen atmosphere using *Schlenk* techniques. Commercially available reagents were purchased from *Acros Organics*, *Alfa Aesar*, *BACHEM*, *Sigma Aldrich*, *TCI*, and used without further purification. All solvents were purified by distillation prior to use. Anhydrous DMF, THF and DCM were obtained by a solvent purification system (*PureSolv MD 5* by *Inert*) or purchased from *Sigma Aldrich*. Distilled water was used for aqueous workup. Analytical thin layer chromatography was performed on aluminium plates coated with silica gel. The plates were stained with aqueous $KMnO_4$ solution (1.50 g $KMnO_4$, 10.0 g K_2CO_3 and 100 mg NaOH in 200 mL distilled H_2O) for visualization.

Column chromatography was performed on silica gel (*Macherey-Nagel*, Silica 60, particle size 0.040–0.063 mm) in self-packed glass columns. Eluents were used as described in the respective procedure. In some cases, desactivated silica gel was used: Silica gel and 10 wt% K_2CO_3 were suspended in MeOH and the mixture was heated under reflux for 6 h. The mixture was filtered and the silica gel was thoroughly washed with methanol and dried under reduced pressure.

NMR spectra were measured at room temperature on a *Bruker* AVANCE III 400 FT-NMR spectrometer. Chemical shifts are reported in parts per million (ppm, δ-scale) referring to the residual solvent peak (^{1}H: $CDCl_3$: δ = 7.26 ppm, CD_3OD: δ = 3.31 ppm; ^{13}C: $CDCl_3$: δ = 77.1 ppm, CD_3OD: δ = 49.0 ppm). Analysis followed first order and data is reported in the following order: chemical shift (in ppm), multiplicity (s = singlet, d = doublet, dd = doublet of doublets, br dd = broad doublet of doublets, ddt = doublet of doublets of triplets, dt = doublet of triplets, t = triplet, td = triplet of doublets, q = quartet, m = multiplet, br m = broad multiplet), coupling constant(s) (in Hz), integration. The stated atom numbers of C-/H-atoms for the attribution of NMR signals are not conform to IUPAC nomenclature and were chosen for clarity reasons. Signal assignment was accomplished with additional information from DEPT135, ^{1}H,^{1}H-COSY, ^{1}H,^{13}C-HSQC and ^{1}H,^{13}C-HMBC measurements.

Mass spectra (for structure confirmation after chemical synthesis) were recorded with a *Bruker Daltonics micrOTOF* and *micrOTOF-Q* III spectrometer by electrospray ionization (ESI). Information on mass-spectrometry in the context of lipidomics see below.

Chemical Synthesis

tert-Butyl-((2S,3R,E)-18-azido-1-((tert-butyldimethylsilyl)oxy)-3-(methoxymethoxy)octadec-4-en-2-yl)-carbamate (**5**)

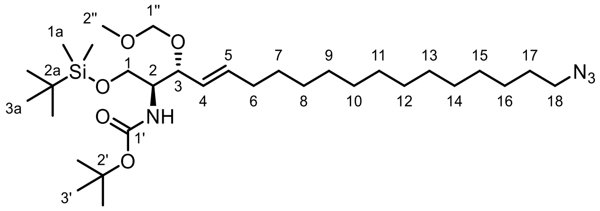

The starting material of this reaction was prepared in a multi-step synthesis according to a literature procedure.[1]

tert-Butyl-((2S,3R,E)-18-azido-1-((*tert*-butyldimethylsilyl)oxy)-3-hydroxyoctadec-4-en-2-yl)-carbamate (1.39 g, 2.50 mmol, 1.00 equiv.) was dissolved in anh. DCM (20 mL). The solution was cooled to 0 °C and MOM-Cl (3.81 mL, 50.1 mmol, 20.0 equiv.) and DIPEA (8.52 mL, 50.1 mmol, 20.0 equiv.) were added. The solution was stirred for 4 h, and then diluted with DCM (40 mL). The organic phase was washed with sat. aq. $NaHCO_3$ solution (40 mL), sat. aq. NH_4Cl solution (30 mL), brine (30 mL) and dried over $MgSO_4$. The solvent was removed under reduced pressure and the crude product was purified by column chromatography (CH/EtOAc = 25/1) to yield the title compound (1.28 g, 2.14 mmol, 85%) as a highly viscous colorless oil.

^{1}H NMR (400 MHz, $CDCl_3$): δ = 5.69 (dt, ^3J = 15.2 Hz, ^3J = 6.8 Hz, 1H, *H*-5), 5.32 (dd, ^3J = 15.2 Hz, ^3J = 8.3 Hz, 1H, *H*-4), 4.69 (d, ^2J = 6.5 Hz, 1H, *H*-1'', superimposes N*H* signal), 4.51 (d, ^2J = 6.5 Hz, 1H, *H*-1''), 4.07 (br dd, ^3J = 7.7 Hz, ^3J = 7.7 Hz, 1H, *H*-3), 3.88–3.80 (m, 1H, *H*-1), 3.72–3.61 (m, 2H, *H*-2, *H*-1), 3.35 (s, 3H, *H*-2''), 3.25 (t, ^3J = 7.0 Hz, 2H, *H*-18), 2.03 (td, ^3J = 6.8 Hz, ^3J = 6.8 Hz, 2H, *H*-6), 1.64–1.55 (m, 2H, *H*-17), 1.42 (s, 9H, *H*-3'), 1.38–1.23 (m, 20H, *H*-7–16), 0.89 (s, 9H, *H*-3a), 0.05 (2 × s, 6H, *H*-1a) ppm; **^{13}C NMR** (100 MHz, $CDCl_3$): δ = 155.5 (*C*-1'), 137.0 (*C*-5), 126.8 (*C*-4), 93.6 (*C*-1''), 79.1 (*C*-2'), 76.0 (*C*-3), 61.7 (*C*-1), 55.6 (*C*-2''), 54.9 (*C*-2), 51.6 (*C*-18), 32.4 (*C*-6), 28.9 (*C*-17), 28.5 (3C, *C*-3'), 29.73, 29.69, 29.67, 29.62, 29.56, 29.3, 29.24, 29.20, 26.8 (10C, *C*-7–16), 26.0 (3C, *C*-3a), 18.3 (*C*-2a), -5.3, -5.4 (2C, *C*-1a) ppm; **HRMS** (ESI+): *m/z* calcd for $C_{31}H_{62}N_4NaO_5Si$ $[M+Na]^+$ 621.4382, found 621.4368, $|\Delta m/z|$ = 2.2 ppm.

tert-Butyl-((2S,3R,E)-18-azido-1-(hydroxy-3-(methoxymethoxy)octadec-4-en-2-yl)-carbamate (**7**)

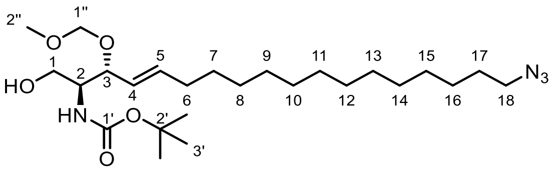

tert-Butyl-((2S,3R,E)-18-azido-1-((*tert*-butyldimethylsilyl)oxy)-3-(methoxymethoxy)octadec-4-en-2-yl)-carbamate (**5**, 253 mg, 422 µmol, 1.00 equiv.) was dissolved in anh. THF (9 mL) and the solution was cooled to 0 °C. TBAF solution (1 M in THF, 591 µL, 591 µmol, 1.40 equiv.) was added dropwise and the solution was stirred for 2 h. Then, H_2O (50 mL) was added and the aqueous phase was extracted with EtOAc (3 × 50 mL). The combined organic layers were washed with brine (50 mL) and dried over $MgSO_4$. The solvent was removed under reduced pressure and the crude product was purified by column chromatography (CH/EtOAc = 4/1) to yield the title compound (182 mg, 411 µmol, 97%) as a colorless oil.

^{1}H NMR (400 MHz, $CDCl_3$): δ = 5.75 (dt, ^3J = 15.4 Hz, ^3J = 7.0 Hz, 1H, *H*-5), 5.34 (ddt, ^3J = 15.5 Hz, ^3J = 8.0 Hz, ^4J = 1.3 Hz, 1H, *H*-4), 5.23 (d, ^3J = 7.2 Hz, 1H, N*H*), 4.66 (d, ^2J = 6.6 Hz, 1H, *H*-1''), 4.52 (d, ^2J = 6.6 Hz, 1H, *H*-1''), 4.23 (dd, ^3J = 7.7 Hz, ^3J = 4.7 Hz, 1H, *H*-3), 3.94 (dd, ^2J = 11.3 Hz, ^3J = 3.5 Hz, 1H, *H*-1), 3.66 (dd, ^2J = 11.5 Hz, ^3J = 3.3 Hz, 1H, *H*-1), 3.66–3.59 (m, 1H, superimposes *H*-1 signal, *H*-2), 3.37 (s, 3H, *H*-2''), 3.25 (t, ^3J = 7.0 Hz, 2H, *H*-18), 2.04 (dt, ^3J = 6.8 Hz, ^3J = 6.8 Hz, 2H, *H*-6), 1.63–1.54 (m, 2H, *H*-17), 1.44 (s, 9H, *H*-3'), 1.39–1.24 (m, 20H, *H*-7–16) ppm; **^{13}C NMR** (100 MHz, $CDCl_3$): δ = 156.0 (*C*-1'), 137.1 (*C*-5), 126.0 (*C*-4), 93.9 (*C*-1''), 79.6 (*C*-2'), 78.6 (*C*-3), 62.5 (*C*-1), 55.8 (*C*-2''), 55.0 (*C*-2), 51.6 (*C*-18), 32.4 (*C*-6), 28.9 (*C*-17), 28.4 (3C, *C*-3'), 29.70, 29.67, 29.65, 29.60, 29.55, 29.5, 29.2, 29.1, 26.8 (10C, *C*-7–16) ppm; **HRMS** (ESI+): *m/z* calcd for $C_{25}H_{48}N_4NaO_5$ 507.3517, found 507.3500, |Δ*m/z*| = 3.3 ppm.

tert-Butyl-((2S,3R,E)-18-azido-1-(((2-bromoethoxy)(hydroxy)phosphoryl)oxy)-3-(methoxy-methoxy)-octadec-4-en-2-yl)carbamate (**9**)

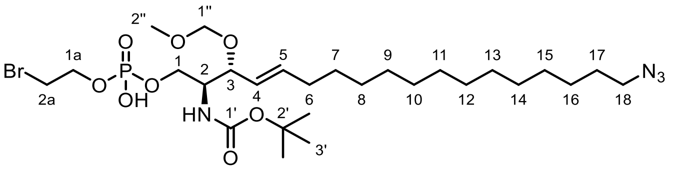

The introduction of the phosphate ester was performed following a modified procedure by *Pinkert et al.*[2]

β-Bromoethylphosphoryl dichloride (11.0 µL, 85.8 µmol, 4.00 equiv.) was dissolved in anh. DCM (0.3 mL) and the solution was cooled to 0 °C. Pyridine (10.4 µL, 129 µmol, 6.00 equiv.) was added and the mixture was stirred for 5 min. Then, *tert*-butyl ((2S,3R,E)-18-azido-1-hydroxy-3-(methoxymethoxy)octadec-4-en-2-yl)carbamate (**7**, 10.4 mg, 21.5 µmol, 1.00 equiv.), dissolved in anh. DCM (0.3 mL), was added dropwise and the reaction was stirred for further 4 h at 0 °C. Sat. aq. $NaHCO_3$ solution (2 mL) was added and the solution was stirred vigorously at rt for 20 min. $CHCl_3$ (20 mL), MeOH (5 mL) and H_2O (15 mL) were added. The organic phase was separated and the aqueous phase was extracted with $CHCl_3$ (20 mL). The combined organic layers were dried over $MgSO_4$ and the crude product was purified by column chromatography (DCM/MeOH = 15/1 → 10/1 → 4/1) to yield the title compound (11.8 mg, 17.6 µmol, 82%) as a colorless waxy solid.

^{1}H NMR (400 MHz, CD_3OD): δ = 5.74 (dt, ^3J = 15.1 Hz, ^3J = 6.8 Hz, 1H, *H*-5), 5.33 (ddt, ^3J = 15.4 Hz, ^3J = 8.7 Hz, ^4J = 1.4 Hz, 1H, *H*-4), 4.68 (d, ^2J = 6.6 Hz, 1H, *H*-1''), 4.51 (d, ^2J = 6.6 Hz, 1H, *H*-1''), 4.20–4.12 (m, 2H, *H*-1a), 4.11–4.02 (m, 2H, *H*-1, *H*-3), 4.02–3.93 (m, 1H, *H*-1), 3.81–3.73 (m, 1H, *H*-2), 3.59 (t, ^3J = 6.3 Hz, 2H, *H*-2a), 3.36 (s, 3H, *H*-2''), 3.28 (t, ^3J = 6.8 Hz, 2H, *H*-18), 2.07 (td, ^3J = 6.8 Hz, ^3J = 6.7 Hz, 2H, *H*-6), 1.63–1.54 (m, 2H, *H*-17), 1.44 (s, 9H, *H*-3'), 1.41–1.28 (m, 20H, *H*-7–16) ppm; **^{13}C NMR** (100 MHz, CD_3OD): δ = 158.0 (*C*-1'), 138.5 (*C*-5), 127.8 (*C*-4), 94.6 (*C*-1''), 80.2 (*C*-2'), 77.7 (*C*-3), 66.7 (d, $J_{C\text{-}P}$ = 5.0 Hz, *C*-1a), 65.8 (d, $J_{C\text{-}P}$ = 5.2 Hz, *C*-1), 56.1 (*C*-2''), 55.6 (d, $J_{C\text{-}P}$ = 7.8 Hz, *C*-2), 52.4 (*C*-18), 33.5 (*C*-6), 32.0 (d, $J_{C\text{-}P}$ = 8.1 Hz, *C*-2a), 30.77, 30.74, 30.71, 30.67, 30.65, 30.63, 30.33, 30.29, 27.8 (10C, *C*-7–16), 29.9 (*C*-17), 28.9 (3C, *C*-3') ppm; **HRMS** (ESI–): *m/z* calcd for $C_{27}H_{51}BrN_4O_8P$ $[M\text{-}H]^-$ 669.2633, found 669.2635, $|\Delta m/z|$ = 0.2 ppm.

(2S,3R,E)-18-Azido-2-((tert-butoxycarbonyl)amino)-3-(methoxymethoxy)octadec-4-en-1-yl (2-(dimethyl(prop-2-yn-1-yl)ammonio)ethyl) phosphate (**11**)

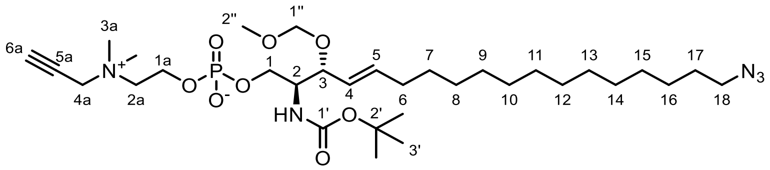

tert-Butyl-((2S,3R,E)-18-azido-1-(((2-bromoethoxy)(hydroxy)phosphoryl)oxy)-3-(methoxymethoxy)octadec-4-en-2-yl)carbamate (**9**, 11.8 mg, 17.6 µmol, 1.00 equiv.) was dissolved in $CHCl_3$ (0.3 mL), MeCN (0.3 mL) and iPrOH (0.3 mL). *N*,*N*-Dimethylprop-2-yn-1-amine (0.3 mL) was added and the reaction mixture was stirred for 20 h at rt, then for 21 h at 45 °C. The solvent was removed under reduced pressure and the crude product was purified by column chromatography ($CHCl_3$/MeOH/H_2O = 55/45/3) to yield the title compound (7.40 mg, 11.0 µmol, 63%) as a colorless waxy solid.

^{1}H NMR (400 MHz, CD_3OD): δ = 5.74 (dt, ^3J = 15.2 Hz, ^3J = 6.8 Hz, 1H, *H*-5), 5.32 (ddt, ^3J = 15.3 Hz, ^3J = 8.6 Hz, ^4J = 1.4 Hz, 1H, *H*-4), 4.68 (d, ^2J = 6.6 Hz, 1H, *H*-1''), 4.51 (d, ^2J = 6.6 Hz, 1H, *H*-1''), 4.44 (d, ^4J = 2.4 Hz, 2H, *H*-4a), 4.33–4.25 (m, 2H, *H*-1a), 4.10–3.96 (m, 3H, *H*-1, *H*-3), 3.78–3.68 (m, 3H, *H*-2, *H*-2a), 3.57 (t, ^4J = 2.5 Hz, 1H, *H*-6a), 3.36 (s, 3H, *H*-2''), 3.28 (s, 6H, *H*-3a), 3.28 (t, ^3J = 6.8 Hz, 2H, *H*-18, superimposed by H-3a signal), 2.07 (dt, ^3J = 6.8 Hz, ^3J = 6.8 Hz, 2H, *H*-6), 1.64–1.54 (m, 2H, *H*-17), 1.43 (s, 9H, *H*-3'), 1.40–1.27 (m, 20H, *H*-7–16) ppm; **^{13}C NMR** (100 MHz, CD_3OD): δ = 158.0 (*C*-1'), 138.6 (*C*-5), 127.9 (*C*-4), 94.5 (*C*-1''), 83.4* (*C*-5a), 80.1 (*C*-2'), 77.6 (*C*-3), 72.0* (*C*-6a), 65.9 (d, $J_{C\text{-}P}$ = 5.4 Hz, *C*-1), 65.3 (d, $J_{C\text{-}P}$ = 7.3 Hz, *C*-2a), 60.2 (d, $J_{C\text{-}P}$ = 5.0 Hz, *C*-1a), 56.4 (*C*-4a), 56.1 (*C*-2''), 55.6 (d, $J_{C\text{-}P}$ = 7.9 Hz, *C*-2), 52.4 (*C*-18), 52.0 (2C, *C*-3a), 33.4 (*C*-6), 30.76, 30.73, 30.70, 30.66, 30.64, 30.61, 30.32, 30.28, 30.27, 27.8 (10C, *C*-7–16), 29.9 (*C*-17), 28.9 (3C, *C*-3') ppm; **HRMS** (ESI+): *m/z* calcd for $C_{32}H_{60}N_5NaO_8P$ $[M+Na]^+$ 696.4072, found 696.4075, |Δ*m/z*| = 0.5 ppm.

**H*-6a is exchanged by deuterium. This leads to a very low intensity of the alkyne signals of *C*-5a and *C*-6a. The signal of *H*-6a is only detected upon immediate measurement after dissolving the sample in CD_3OD.

Perfluorophenyl (S)-2-((tert-butoxycarbonyl)amino)hexanoate (**13**)

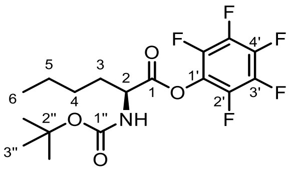

N-Boc-L-norleucin (200 mg, 865 µmol, 1.00 equiv.) and pentafluorophenol (239 mg, 1.30 mmol, 1.50 equiv.) were dissolved in anh. DCM (8 mL). The solution was cooled to 0 °C and EDC hydrochloride (249 mg, 1.30 mmol, 1.50 equiv.) and DMAP (10.6 mg, 86.5 µmol, 0.10 equiv.) were added. The reaction mixture was warmed to rt and stirred for 16 h. The mixture was diluted with DCM (40 mL) and the organic phase was washed with HCl solution (1 M, 2 × 40 mL), then with sat. aq. $NaHCO_3$ solution (2 × 40 mL). The organic layer was dried over $MgSO_4$ and the solvent was removed under reduced pressure. The crude product was purified by column chromatography (CH/EtOAc = 20/1) to afford the title compound (212 mg, 533 µmol, 62%) as a colorless oil.

^{1}H NMR (400 MHz, $CDCl_3$): δ = 4.99 (d, ^{3}J = 8.1 Hz, 1H, N*H*), 4.73–4.56 (m, 1H, *H*-2), 2.04–1.90 (m, 1H, *H*-3), 1.89–1.74 (m, 1H, *H*-3), 1.47 (s, 9H, *H*-3''), 1.45–1.35 (m, 4H, H-4–5), 0.94 (t, ^{3}J = 7.1 Hz, 3H, *H*-6) ppm; **^{13}C NMR** (100 MHz, $CDCl_3$): δ = 169.4 (*C*-1), 155.2 (*C*-1''), 141.1 (m, 2C, C_{ar}), 139.7 (m, C_{ar}), 138.0 (m, 2C, C_{ar}), 124.9 (m, C_{ar}), 80.6 (*C*-2''), 53.6 (*C*-2), 32.0 (*C*-3), 28.3 (3C, *C*-3''), 27.3, 22.3 (*C*-4–5), 13.8 (*C*-6) ppm; **HRMS** (ESI+): *m/z* calcd for $C_{17}H_{20}F_5NNaO_4$ $[M+Na]^+$ 420.1205, found 420.1213, $|\Delta m/z|$ = 1.9 ppm.

(2S,3R,E)-18-Azido-2-((S)-2-((tert-butoxycarbonyl)amino)hexanamido)-3-hydroxyoctadec-4-en-1-yl (2-(dimethyl(prop-2-yn-1-yl)ammonio)ethyl) phosphate (**15**)

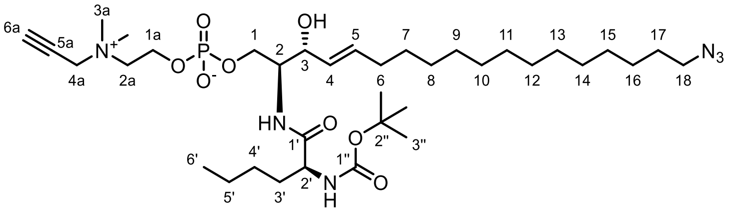

(2S,3R,E)-18-Azido-2-((*tert*-butoxycarbonyl)amino)-3-(methoxymethoxy)octadec-4-en-1-yl (2-(dimethyl(prop-2-yn-1-yl)ammonio)ethyl) phosphate (**11**, 23.1 mg, 34.3 µmol, 1.00 equiv.) was dissolved in anh. MeOH (2 mL). HCl solution (4 M in 1,4-dioxane, 214 µL, 857 µmol, 25.0 equiv.) was added and the solution was stirred for 2 h at rt. The solution was purged with nitrogen for 10 min and the solvent was removed under reduced pressure. TLC and crude ^{1}H NMR confirmed successful deprotection of the MOM and Boc group. The crude product was subjected to the following reaction conditions without further purification.

Crude (2S,3R,E)-2-amino-18-azido-3-hydroxyoctadec-4-en-1-yl-(2-(dimethyl(prop-2-yn-1-yl)-ammonio)ethyl) phosphate and pentafluorophenyl (S)-2-((tert-butoxycarbonyl)amino)-hexanoate (**13**, 20.4 mg, 51.4 µmol, 1.50 equiv.) were dissolved in anh. DCM (2 mL). DIPEA (11.9 mL, 68.6 µmol, 2.00 equiv.) was added and the solution was stirred for 18 h at rt. The solvent was removed under reduced pressure and the crude product was purified by column chromatography on desactivated silica gel ($CHCl_3$/MeOH/H_2O = 85/15/1) to yield the title compound (21.6 mg, 29.1 µmol, 85%) as a colorless oil.

^{1}H NMR (400 MHz, CD_3OD): δ = 7.84 (d, ^{3}J = 8.9 Hz, N*H*), 6.65 (d, ^{3}J = 8.3 Hz, N*H*), 5.72 (dt, ^{3}J = 15.2 Hz, ^{3}J = 6.8 Hz, 1H, *H*-5), 5.45 (dd, ^{3}J = 15.3 Hz, ^{3}J = 7.3 Hz, 1H, *H*-4), 4.44 (d, ^{4}J = 2.3 Hz, 2H, *H*-4a), 4.36–4.25 (m, 2H, *H*-1a), 4.18–4.00 (m, 3H, *H*-1, *H*-3, *H*-2'), 4.00–3.89 (m, 2H, *H*-1, *H*-2), 3.79–3.70 (m, 2H, *H*-2a), 3.56 (t, ^{4}J = 2.5 Hz, 1H, *H*-6a), 3.28 (s, 6H, *H*-3a), 3.28 (t, ^{3}J = 6.8 Hz, 2H, *H*-18, signal superimposed by *H*-3a signal), 2.03 (td, ^{3}J = 7.2 Hz, ^{3}J = 6.9 Hz, 2H, *H*-6), 1.80–1.67 (m, 1H, *H*-3'), 1.64–1.50 (m, 3H, *H*-17, *H*-3'), 1.45 (s, 9H, *H*-3''), 1.42–1.26 (m, 24H, *H*-7–16, *H*-4'–5'), 0.93 (t, ^{3}J = 6.5 Hz, 3H, *H*-6') ppm; **^{13}C NMR** (100 MHz, CD_3OD): δ = 175.0 (*C*-1'), 157.7 (*C*-1''), 135.2 (*C*-5), 130.9 (*C*-4), 83.2 (C-5a), 80.4 (*C*-2''), 72.4 (C-6a), 72.3 (*C*-3), 65.7 (d, $J_{C\text{-}P}$ = 5.3 Hz, *C*-1), 65.3 (d, $J_{C\text{-}P}$ = 7.5 Hz, *C*-2a), 60.3 (d, $J_{C\text{-}P}$ = 4.8 Hz, *C*-1a), 56.4 (*C*-4a), 56.1 (*C*-2'), 55.3 (d, $J_{C\text{-}P}$ = 7.5 Hz, *C*-2), 52.4 (*C*-18), 52.0 (2C, *C*-3a), 33.9 (*C*-3'), 33.5 (*C*-6), 30.79, 30.74, 30.68, 30.6, 30.5, 30.28, 30.25, 29.2, 27.8, 23.5 (12C, *C*-7–16, *C*-4'–5'), 29.9 (*C*-17), 28.8 (3C, *C*-3''), 14.4 (*C*-6) ppm; **HRMS** (ESI+): *m/z* calcd for $C_{36}H_{67}N_6NaO_8P$ $[M+Na]^+$ 765.4650, found 765.4669, $|\Delta m/z|$ = 2.4 ppm.

(2S,3R,E)-2-((S)-2-ammoniohexanamido)-18-azido-3-hydroxyoctadec-4-en-1-yl-(2-(dimethyl(prop-2-yn-1-yl)ammonio)ethyl) phosphate 2,2,2-trifluoroacetate (**TFSM 1**)

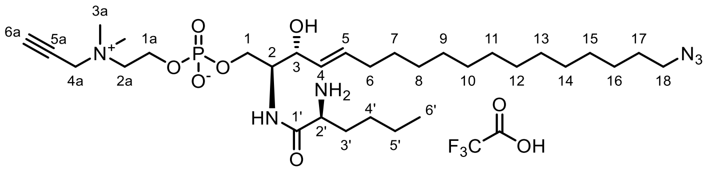

(2S,3R,E)-18-azido-2-((S)-2-((*tert*-butoxycarbonyl)amino)hexanamido)-3-hydroxyoctadec-4-en-1-yl (2-(dimethyl(prop-2-yn-1-yl)ammonio)ethyl) phosphate (**15**, 4.70 mg, 6.33 µmol) was dissolved in DCM (1.5 mL). The solution was cooled to 0 °C and TFA (0.5 mL) was added. The reaction mixture was stirred for 2 h at this temperature. The solvent was removed under reduced pressure to yield the title compound (quant., TFA salt) as a colorless oil.

^{1}H NMR (400 MHz, CD_3OD): δ = 8.33 (d, ^{3}J = 9.0 Hz, N*H*), 5.76 (dt, ^{3}J = 15.0 Hz, ^{3}J = 6.8 Hz, 1H, *H*-5), 5.45 (ddt, ^{3}J = 15.3 Hz, ^{3}J = 7.7 Hz, ^{4}J = 1.4 Hz, 1H, *H*-4), 4.44 (d, ^{4}J = 2.4 Hz, 2H, *H*-4a), 4.34–4.27 (m, 2H, *H*-1a), 4.14–4.01 (m, 3H, *H*-1, *H*-3), 4.01–3.91 (m, 1H, *H*-2), 3.79 (dd, ^{3}J = 6.8 Hz, ^{3}J = 6.8 Hz, 1H, *H*-2‘), 3.77–3.72 (m, 2H, *H*-2a), 3.57 (t, ^{4}J = 2.5 Hz, 1H, *H*-6a), 3.28 (s, 6H, *H*-3a), 3.28 (t, ^{3}J = 6.8 Hz, 2H, *H*-18, signal superimposed by *H*-3a signal), 2.04 (td, ^{3}J = 7.0 Hz, ^{3}J = 7.0 Hz, 2H, *H*-6), 1.88–1.67 (m, 2H, *H*-3‘), 1.63–1.54 (m, 2H, *H*-17), 1.43–1.29 (m, 24H, *H*-7–16, H-4‘–5‘), 0.96 (t, ^{3}J = 6.9 Hz, 3H, *H*-6‘) ppm; **^{13}C NMR** (100 MHz, CD_3OD): δ = 170.1 (*C*-1‘), 135.7 (*C*-5), 131.1 (*C*-4), 83.2 (C-5a), 72.4 (C-6a), 72.0 (*C*-3), 65.8 (d, $J_{C\text{-}P}$ = 5.7 Hz, *C*-1), 65.3 (d, $J_{C\text{-}P}$ = 7.7 Hz, *C*-2a), 60.3 (d, $J_{C\text{-}P}$ = 5.0 Hz, *C*-1a), 56.4 (*C*-4a), 55.7 (d, $J_{C\text{-}P}$ = 6.6 Hz, *C*-2), 54.7 (*C*-2‘), 52.4 (*C*-18), 52.0 (2C, *C*-3a), 33.5 (*C*-6), 32.4 (*C*-3’), 29.9 (*C*-17), 30.8, 30.73, 30.66, 30.65, 30.6, 30.5, 30.3, 28.1, 27.8, 23.5 (12C, *C*-7–16, *C*-4’–5’), 14.1 (*C*-6’) ppm; **HRMS** (ESI+): *m/z* calcd for $C_{31}H_{60}N_6O_6P$ $[M+H]^+$ 643.4312, found 643.4343, |Δ*m/z*| = 4.8 ppm.

tert-Butyl-((2S,3R,E)-1-((*tert*-butyldimethylsilyl)oxy)-3-(methoxymethoxy)octadec-4-en-2-yl)-carbamate (**6**)

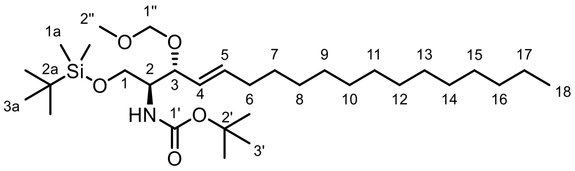

The starting material of this reaction was prepared in a multi-step synthesis according to a literature procedure.[3]

tert-Butyl ((2S,3R,E)-1-((tert-butyldimethylsilyl)oxy)-3-hydroxyoctadec-4-en-2-yl)carbamate (1.27 g, 2.47 mmol, 1.00 equiv.) was dissolved in anh. DCM (20 mL) and the solution was cooled to 0 °C. MOM-Cl (3.75 mL, 49.4 mmol, 20.0 equiv.) was added, followed by the dropwise addition of anh. DIPEA (8.41 mL, 49.4 mmol, 20.0 equiv.). The solution was allowed to warm to rt and stirred for 4 h. The reaction mixture was diluted with DCM (30 mL), washed with sat. aq. $NaHCO_3$ solution (30 mL), sat. aq. NH_4Cl solution (30 mL) and brine (30 mL). The organic layer was dried over $MgSO_4$ and the solvent was removed under reduced pressure. The crude product was purified by column chromatography (CH/EtOAc = 25/1) to afford the title compound (1.02 g, 1.82 mmol, 74%) as a colorless oil.

^{1}H NMR (400 MHz, $CDCl_3$): δ = 5.68 (dt, ^3J = 15.2 Hz, ^3J = 6.8 Hz, 1H, *H*-5), 5.32 (dd, ^3J = 15.4 Hz, ^3J = 8.5 Hz, 1H, *H*-4), 4.69 (d, ^2J = 6.6 Hz, 1H, *H*-1'' and m, 1H, N*H*), 4.50 (d, ^2J = 6.6 Hz, 1H, *H*-1''), 4.13–3.99 (m, 1H, *H*-3), 3.90–3.77 (m, 1H, *H*-1), 3.74–3.52 (m, 2H, *H*-2, *H*-1), 3.34 (s, 3H, *H*-2''), 2.03 (td, ^3J = 6.8 Hz, ^3J = 6.8 Hz, 2H, *H*-6), 1.42 (s, 9H, *H*-3'), 1.39–1.18 (m, 22H, *H*-7–17), 0.89 (s, 9H, *H*-3a), 0.87 (t, ^3J = 7.0 Hz, 3H, *H*-18), 0.05 (2 × s, 6H, *H*-1a) ppm; **^{13}C NMR** (100 MHz, $CDCl_3$): δ = 155.5 (*C*-1'), 137.1 (*C*-5), 126.7 (*C*-4), 93.6 (*C*-1''), 79.1 (*C*-2'), 76.0 (*C*-3), 61.7 (*C*-1), 55.6 (*C*-2''), 54.9 (*C*-2), 32.4 (*C*-6), 32.0, 29.77, 29.76, 29.74, 29.69, 29.6, 29.4, 29.3, 29.2, 22.8 (11C, *C*-7–17), 28.5 (3C, *C*-3'), 26.0 (3C, *C*-3a), 18.3 (*C*-2a), 14.2 (*C*-18), –5.3, –5.4 (2C, *C*-1a) ppm; **HRMS** (ESI+): *m/z* calcd for $C_{31}H_{63}NNaO_5Si$ $[M+Na]^+$ 580.4368, found 580.4377, |Δ*m/z*| = 1.6 ppm.

tert-Butyl ((2S,3R,E)-1-hydroxy-3-(methoxymethoxy)octadec-4-en-2-yl)carbamate (**8**)

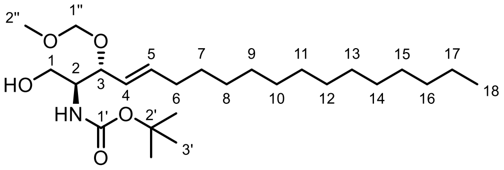

tert-Butyl-((2S,3R,E)-1-((*tert*-butyldimethylsilyl)oxy)-3-(methoxymethoxy)octadec-4-en-2-yl)-carbamate (**6**, 966 mg, 1.73 mmol, 1.00 equiv.) was dissolved in anh. THF (35 mL) and the solution was cooled to 0 °C. TBAF solution (1 M in THF, 2.08 mL, 2.08 mmol, 1.20 equiv.) was added and the mixture was stirred for 30 min at 0 °C and for further 30 min at rt. H_2O (30 mL) was added and the aqueous phase was extracted with EtOAc (3 × 50 mL). The combined organic layers were washed with brine and dried over $MgSO_4$. The solvent was removed under reduced pressure and the crude product was purified by column chromatography (CH/EtOAc = 4/1) to afford the title compound (602 mg, 1.36 mmol, 78%) as a waxy white solid.

^{1}H NMR (400 MHz, $CDCl_3$): δ = 5.75 (dt, ^3J = 15.4 Hz, ^3J = 6.8 Hz, 1H, *H*-5), 5.34 (ddt, ^3J = 15.5 Hz, ^3J = 7.9 Hz, ^4J = 1.4 Hz, 1H, *H*-4), 5.23 (d, ^3J = 7.5 Hz, 1H, N*H*), 4.66 (d, ^2J = 6.6 Hz, 1H, *H*-1''), 4.51 (d, ^2J = 6.6 Hz, 1H, *H*-1''), 4.23 (dd, ^3J = 7.7 Hz, ^3J = 4.8 Hz, 1H, *H*-3), 3.98–3.88 (m, 1H, *H*-1), 3.70–3.58 (m, 2H, *H*-1, *H*-2), 3.37 (s, 3H, *H*-2''), 2.77 (br d, ^3J = 6.9 Hz, 1H, O*H*), 2.04 (td, ^3J = 6.8 Hz, ^3J = 6.8 Hz, 2H, *H*-6), 1.44 (s, 9H, *H*-3'), 1.37–1.22 (m, 22H, *H*-7–17), 0.87 (t, ^3J = 6.9 Hz, 3H, *H*-18) ppm; **^{13}C NMR** (100 MHz, $CDCl_3$): δ = 156.0 (*C*-1'), 137.1 (*C*-5), 126.0 (*C*-4), 93.9 (*C*-1''), 79.6 (*C*-2'), 78.6 (*C*-3), 62.5 (*C*-1), 55.8 (*C*-2''), 55.0 (*C*-2), 32.4 (*C*-6), 32.0, 29.76, 29.74, 29.73, 29.67, 29.5, 29.4, 29.2, 29.1, 22.8 (11C, *C*-7–17), 28.4 (3C, *C*-3'), 14.2 (*C*-18) ppm; **HRMS** (ESI+): *m/z* calcd for $C_{25}H_{49}NNaO_5$ $[M+Na]^+$ 466.3503, found 466.3508, $|\Delta m/z|$ = 1.0 ppm.

The spectroscopic data agree with those reported in the literature.[2]

tert-Butyl-((2S,3R,E)-1-(((2-bromoethoxy)(hydroxy)phosphoryl)oxy)-3-(methoxy-methoxy)-octadec-4-en-2-yl)carbamate (**10**)

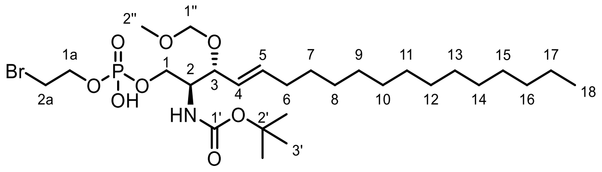

β-Bromoethylphosphoryl dichloride (643 µL, 5.02 mmol, 4.00 equiv.) was dissolved in anh. DCM (20 mL) and the solution was cooled to 0 °C. Pyridine (607 µL, 7.53 mmol, 6.00 equiv.) was added and the mixture was stirred for 5 min. Then, a solution of *tert*-Butyl ((2S,3R,E)-1-hydroxy-3-(methoxymethoxy)octadec-4-en-2-yl)carbamate (**8**, 557 mg, 1.26 mmol, 1.00 equiv.) in anh. DCM (15 mL) was added and the reaction was stirred for 4.5 h. Sat. aq. $NaHCO_3$ solution (50 mL) was added and the biphasic mixture was stirred vigorously for 30 min. The mixture was diluted with H_2O (30 mL) and brine and the aqueous phase was extracted with $CHCl_3$ (4 × 75 mL). The combined organic layers were washed with brine and dried over $MgSO_4$. The solvent was removed under reduced pressure and the crude product was purified by column chromatography (DCM/MeOH = 30/1 → 10/1) to afford the title compound (444 mg, 704 µmol, 56%) as a colorless oil.

^{1}H NMR (400 MHz, CD_3OD): δ = 5.74 (dt, ^3J = 15.1 Hz, ^3J = 6.9 Hz, 1H, *H*-5), 5.33 (dd, ^3J = 15.4 Hz, ^3J = 8.6 Hz, 1H, *H*-4), 4.68 (d, ^2J = 6.6 Hz, 1H, *H*-1''), 4.51 (d, ^2J = 6.6 Hz, 1H, *H*-1''), 4.20–4.11 (m, 2H, *H*-1a), 4.11–4.02 (m, 2H, *H*-1, *H*-3), 4.02–3.92 (m, 1H, *H*-1), 3.81–3.65 (m, 1H, *H*-2), 3.58 (t, ^3J = 6.3 Hz, 2H, *H*-2a), 3.36 (s, 3H, *H*-2''), 2.07 (td, ^3J = 6.8 Hz, ^3J = 6.7 Hz, 2H, *H*-6), 1.43 (s, 9H, *H*-3'), 1.52–1.22 (m, 22H, *H*-7–17), 0.90 (t, ^3J = 6.9 Hz, 3H, *H*-18) ppm; **^{13}C NMR** (100 MHz, CD_3OD): δ = 158.0 (*C*-1'), 138.5 (*C*-5), 127.8 (*C*-4), 94.6 (*C*-1''), 80.2 (*C*-2'), 77.7 (*C*-3), 66.7 (d, $J_{C\text{-}P}$ = 5.1 Hz, *C*-1a), 65.8 (d, $J_{C\text{-}P}$ = 5.6 Hz, *C*-1), 56.1 (*C*-2''), 55.6 (d, $J_{C\text{-}P}$ = 7.9 Hz, *C*-2), 33.5 (*C*-6), 32.0 (d, $J_{C\text{-}P}$ = 8.2 Hz, *C*-2a), 33.1, 30.81, 30.79, 30.78, 30.7, 30.6, 30.5, 30.33, 30.29, 23.8 (11C, *C*-7–17), 28.9 (3C, *C*-3'), 14.5 (*C*-18) ppm; **HRMS** (ESI+): *m/z* calcd for $C_{27}H_{53}BrNNaO_8P$ $[M+Na]^+$ 652.2584, found 652.2597, |Δ*m/z*| = 1.9 ppm.

(2S,3R,E)-2-((*tert*-butoxycarbonyl)amino)-3-(methoxymethoxy)octadec-4-en-1-yl (2-(dimethyl(prop-2-yn-1-yl)ammonio)ethyl) phosphate (**12**)

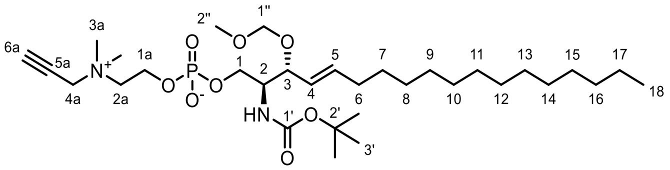

tert-Butyl-((2S,3R,E)-1-(((2-bromoethoxy)(hydroxy)phosphoryl)oxy)-3-(methoxymethoxy)-octadec-4-en-2-yl)carbamate (**10**, 401 mg, 636 µmol, 1.00 equiv.) was dissolved in a mixture of MeCN (4.5 mL), iPrOH (4.5 mL), $CHCl_3$ (4.5 mL) and *N*,*N*-dimethylprop-2-yn-1-amine (4.5 mL) and the solution was stirred for 6 d at rt. The solvent was removed under reduced pressure and the crude product was purified by column chromatography ($CHCl_3$/MeOH/H_2O = 55/45/3) to afford the title compound (279 mg, 441 µmol, 69%) as a white foam.

^{1}H NMR (400 MHz, CD_3OD): δ = 6.74 (d, ^3J = 9.4 Hz, N*H*), 5.74 (dt, ^3J = 15.2 Hz, ^3J = 6.8 Hz, 1H, *H*-5), 5.32 (ddt, ^3J = 15.4 Hz, ^3J = 8.7 Hz, ^4J = 1.3 Hz, 1H, *H*-4), 4.68 (d, ^2J = 6.6 Hz, 1H, *H*-1''), 4.51 (d, ^2J = 6.6 Hz, 1H, *H*-1''), 4.45 (d, ^4J = 2.4 Hz, 2H, *H*-4a), 4.36–4.24 (m, 2H, *H*-1a), 4.10–3.96 (m, 3H, *H*-3, *H*-1), 3.79–3.66 (m, 3H, *H*-2a, *H*-2), 3.57 (t, ^4J = 2.5 Hz, 1H, *H*-6a), 3.36 (s, 3H, *H*-2''), 3.28 (s, 6H, *H*-3a), 2.07 (td, ^3J = 6.8 Hz, ^3J = 6.7 Hz, 2H, *H*-6), 1.43 (s, 9H, *H*-3'), 1.50–1.21 (m, 22H, *H*-7–17), 0.90 (t, ^3J = 6.9 Hz, 3H, *H*-18) ppm; **^{13}C NMR** (100 MHz, CD_3OD): δ = 158.0 (*C*-1'), 138.6 (*C*-5), 127.9 (*C*-4), 94.5 (*C*-1''), 83.2 (*C*-5a), 80.1 (*C*-2'), 77.6 (*C*-3), 72.4 (*C*-6a), 65.9 (d, $J_{C\text{-}P}$ = 5.5 Hz, *C*-1), 65.3 (d, $J_{C\text{-}P}$ = 7.4 Hz, *C*-2a), 60.2 (d, $J_{C\text{-}P}$ = 5.0 Hz, *C*-1a), 56.4 (*C*-4a), 56.1 (*C*-2''), 55.6 (d, $J_{C\text{-}P}$ = 8.0 Hz, *C*-2), 52.0 (2C, *C*-3a), 33.4 (*C*-6), 33.1, 30.80, 30.78, 30.78, 30.7, 30.6, 30.5, 30.33, 30.27, 23.8 (11C, *C*-7–17), 28.9 (3C, *C*-3'), 14.5 (*C*-18) ppm; **HRMS** (ESI+): *m/z* calcd for $C_{32}H_{61}N_2NaO_8P$ $[M+Na]^+$ 655.4058, found 655.4063, |Δ*m/z*| = 0.8 ppm.

Perfluorophenyl N^2-(tert-butoxycarbonyl)-N^6-diazo-*L*-lysinate (**14**)

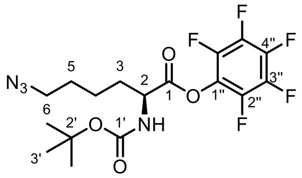

The starting material of this reaction was synthesized according to a protocol by *Yang et al.*[4]

N^2-(*tert*-Butoxycarbonyl)-N^6-diazo-*L*-lysine (154 mg, 566 µmol, 1.00 equiv.) and 2,3,4,5,6-pentafluorophenol (156 mg, 848 µmol, 1.50 equiv.) were dissolved in anh. DCM (4 mL) and the solution was cooled to 0 °C. EDC hydrochloride (163 mg, 848 µmol, 1.50 equiv.) and DMAP (6.91 mg, 56.6 µmol, 0.10 equiv.) were added, the solution was allowed to warm to rt and stirred overnight. DCM (30 mL) was added and the mixture was washed with HCl (1 M, 2 × 20 mL), then with sat. aq. $NaHCO_3$ solution (2 × 20 mL). The organic phase was dried over $MgSO_4$ and the solvent was removed under reduced pressure. The crude product was purified by column chromatography (CH/EtOAc = 20/1) to afford the title compound (160 mg, 365 µmol, 64%) as a colorless oil.

^{1}H NMR (400 MHz, $CDCl_3$): δ = 5.13–4.83 (m, 1H, N*H*), 4.72–4.34 (m, 1H, *H*-2), 3.32 (t, ^3J = 6.6 Hz, 2H, *H*-6), 2.08–1.94 (m, 1H, *H*-3), 1.92–1.78 (m, 1H, *H*-3), 1.74–1.62 (m, 2H, *H*-5), 1.61–1.50 (m, 2H, *H*-4), 1.46 (s, 9H, *H*-3') ppm; **^{13}C NMR** (100 MHz, $CDCl_3$): δ = 169.1 (*C*-1), 155.2 (*C*-1'), 141.1 (m), 139.8 (m), 138.0 (m), 124.8 (m) (6C, *C*-1''–4''), 80.8 (*C*-2'), 53.3 (*C*-2), 51.1 (*C*-6), 32.0 (*C*-3), 28.4 (*C*-5), 28.3 (3C, *C*-3'), 22.6 (*C*-4) ppm; **HRMS** (ESI+): *m/z* calcd for $C_{17}H_{19}F_5N_4NaO_4$ $[M+Na]^+$ 461.1219, found 461.1218, |Δ*m/z*| = 0.0 ppm.

(2S,3R,E)-2-((S)-6-Azido-2-((tert-butoxycarbonyl)amino)hexanamido)-3-hydroxy-octadec-4-en-1-yl (2-(dimethyl(prop-2-yn-1-yl)ammonio)ethyl) phosphate (**16**)

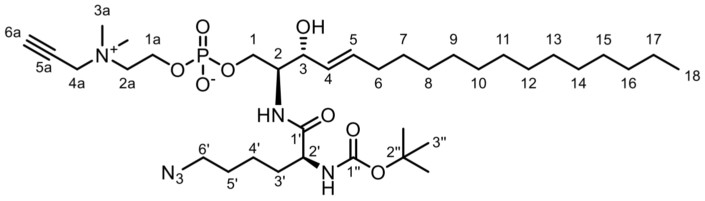

(2S,3R,E)-2-((*tert*-Butoxycarbonyl)amino)-3-(methoxymethoxy)octadec-4-en-1-yl(2-(dimethyl-(prop-2-yn-1-yl)ammonio)ethyl) phosphate (**12**, 17.6 mg, 27.8 µmol, 1.00 equiv.) was dissolved in anh. MeOH (2 mL). HCl solution (4 M in 1,4-dioxane, 0.3 mL) was added and the reaction was stirred for 3.5 h. The solvent was removed under reduced pressure to give the crude amine. Successful deprotection was confirmed by TLC and ^{1}H NMR. The intermediate was immediately subjected to the following reaction conditions without further purification.

The crude amine, perfluorophenyl N^2-(*tert*-butoxycarbonyl)-N^6-diazo-L-lysinate (**14**, 18.3 mg, 41.7 µmol, 1.50 equiv.) and DIPEA (75.0 µL, 55.6 µmol, 2.00 equiv.) were dissolved in anh. DCM (2 mL) and stirred for 3 d at rt. MeOH was added and the solution was stirred for further 2 h. The solvent was removed under reduced pressure and the crude product was purified by column chromatography ($CHCl_3$/MeOH/H_2O/NH_3 (25% aq.) = 85/15/1/0.1%) to afford the title compound (17.5 mg, 23.6 µmol, 85%) as a colorless waxy solid.

^{1}H NMR (400 MHz, CD_3OD): δ = 7.86 (d, ^3J = 9.2 Hz, 1H, N*H*), 5.73 (dt, ^3J = 14.8 Hz, ^3J = 6.9 Hz, 1H, *H*-5), 5.45 (dd, ^3J = 15.4 Hz, ^3J = 7.3 Hz, 1H, *H*-4), 4.45 (d, ^4J = 2.4 Hz, 2H, *H*-4a), 4.37–4.25 (m, 2H, *H*-1a), 4.17–4.02 (m, 3H, *H*-3, *H*-2', *H*-1), 4.02–3.91 (m, 2H, *H*-1, *H*-2), 3.80–3.71 (m, 2H, *H*-2a), 3.56 (t, ^4J = 2.5 Hz, 1H, *H*-6a), 3.36 (m, superimposed by solvent signal, 2H, *H*-6'), 3.29 (s, 6H, *H*-3a), 2.10–1.97 (m, 2H, *H*-6), 1.83–1.69 (m, 1H, *H*-3'), 1.69–1.51 (m, 3H, *H*-3', *H*-5'), 1.45 (s, 9H, *H*-3''), 1.44–1.25 (m, 24H, *H*-7–17, *H*-4'), 0.90 (t, ^3J = 6.8 Hz, 3H, *H*-18) ppm; **^{13}C NMR** (100 MHz, CD_3OD): δ = 174.8 (*C*-1'), 157.7 (*C*-1''), 135.2 (*C*-5), 130.8 (*C*-4), 83.2 (*C*-5a), 80.5 (*C*-2''), 72.4 (*C*-6a), 72.3 (*C*-3), 65.7 (d, J_{C-P} = 5.4 Hz, *C*-1), 65.3 (d, J_{C-P} = 7.5 Hz, *C*-2a), 60.3 (d, J_{C-P} = 4.9 Hz, *C*-1a), 56.5 (*C*-4a), 55.9 (*C*-2'), 55.4 (d, J_{C-P} = 7.8 Hz, *C*-2), 52.3 (*C*-6'), 52.0 (2C, *C*-3a), 33.6 (*C*-3'), 33.5 (*C*-6), 29.6 (*C*-5'), 28.8 (3C, *C*-3''), 33.1, 30.82, 30.80, 30.78, 30.77, 30.67, 30.5, 30.3, 24.2, 23.7 (12C, *C*-7–17, *C*-4'), 14.5 (*C*-18) ppm; **HRMS** (ESI+): *m/z* calcd for $C_{36}H_{67}N_6NaO_8P$ $[M+Na]^+$ 765.4650, found 765.4617, |Δ*m/z*| = 4.4 ppm.

(2S,3R,E)-2-((S)-2-Ammonio-6-azidohexanamido)-3-hydroxyoctadec-4-en-1-yl-(2-(dimethyl-(prop-2-yn-1-yl)ammonio)ethyl) phosphate 2,2,2-trifluoroacetate (**TFSM 2**)

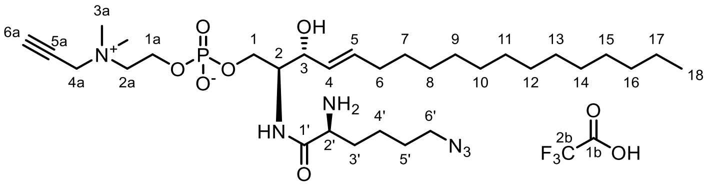

(2S,3R,E)-2-((S)-6-Azido-2-((tert-butoxycarbonyl)amino)hexanamido)-3-hydroxy-octadec-4-en-1-yl (2-(dimethyl(prop-2-yn-1-yl)ammonio)ethyl) phosphate (**16**, 11.0 mg, 14.8 µmol) was dissolved in DCM (1.5 mL) and the solution was cooled to 0 °C. TFA (0.3 mL) was added and the reaction was stirred for 2.5 h. The solvent was removed under reduced pressure and remaining TFA was removed by co-evaporation with MeOH. The product was dried under high-vacuum to afford the title compound (quant., TFA salt) as a colorless oil.

^{1}H NMR (400 MHz, CD_3OD): δ = 5.77 (dt, ^3J = 15.3 Hz, ^3J = 6.6 Hz, 1H, *H*-5), 5.46 (dd, ^3J = 15.3 Hz, ^3J = 7.5 Hz, 1H, *H*-4), 4.45 (d, ^4J = 2.4 Hz, 2H, *H*-4a), 4.40–4.25 (m, 2H, *H*-1a), 4.17–3.88 (m, 4H, *H*-3, *H*-1, *H*-2), 3.82 (t, ^3J = 6.7 Hz, 1H, *H*-2'), 3.79–3.70 (m, 2H, *H*-2a), 3.56 (t, ^4J = 2.5 Hz, 1H, *H*-6a), 3.35 (t, ^3J = 6.7 Hz, 2H, *H*-6'), 3.29 (s, 6H, *H*-3a), 2.12–1.97 (m, 2H, *H*-6), 1.97–1.71 (m, 2H, *H*-3'), 1.69–1.57 (m, 2H, *H*-5'), 1.53–1.43 (m, 2H, *H*-4'), 1.41–1.26 (m, 22H, *H*-7–17), 0.90 (t, ^3J = 6.8 Hz, 3H, *H*-18) ppm; **^{13}C NMR** (100 MHz, CD_3OD): δ = 170.0 (*C*-1'), 163.1 (q, $J_{C\text{-}F}$ = 34.0 Hz, *C*-1b), 135.7 (*C*-5), 131.0 (*C*-4), 118.3 (q, $J_{C\text{-}F}$ = 292.8 Hz, *C*-2b), 83.2 (*C*-5a), 72.4 (*C*-6a), 72.1 (*C*-3), 65.7 (d, $J_{C\text{-}P}$ = 5.7 Hz, *C*-1), 65.2 (d, $J_{C\text{-}P}$ = 7.8 Hz, *C*-2a), 60.3 (d, $J_{C\text{-}P}$ = 4.9 Hz, *C*-1a), 56.4 (*C*-4a), 55.7 (d, $J_{C\text{-}P}$ = 6.7 Hz, *C*-2), 54.5 (*C*-2'), 52.01 (*C*-6'), 52.00 (2C, *C*-3a), 33.5 (*C*-6), 32.2 (*C*-3'), 29.6 (*C*-5'), 33.1, 30.80, 30.79, 30.77, 30.68, 30.50, 30.48, 30.4, 23.7 (11C, *C*-7–17), 23.3 (*C*-4'), 14.4 (*C*-18) ppm; **HRMS** (ESI+): *m/z* calcd for $C_{31}H_{60}N_6O_6P$ $[M+H]^+$ 643.4312, found 643.4314, $|\Delta m/z|$ = 0.3 ppm.

LC-MS/MS Analysis

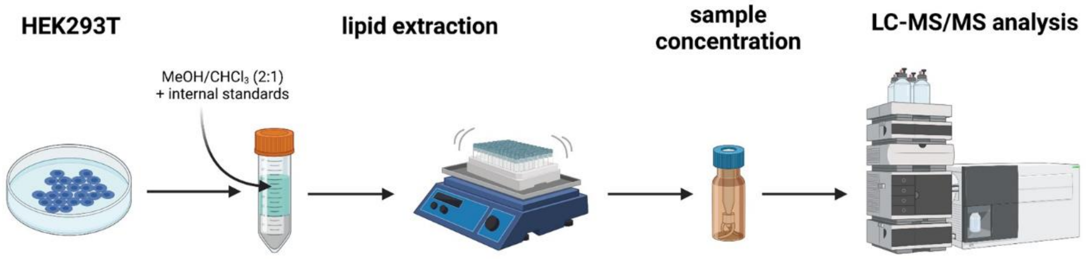

Supplementary Figure 1 Sample preparation workflow for LC-MS/MS identification of TFSM metabolites in HEK293T cells. The experimental set-up included treatment and harvest of cells, lipid extraction in the presence of internal standards, sample concentration by vacuum evaporation, and analysis by liquid chromatography coupled to tandem-mass spectrometry (LC-MS/MS). Supplementary Figure 1 was created with BioRender.com, released under a Creative Commons Attribution-NonCommercial-NoDerivs 4.0 International license.

HEK293T cells were cultured in DMEM (4.5 g/l D-glucose, Sigma-Aldrich, #D6546) supplemented with L-glutamine (4 mM, Sigma-Aldrich, #G7513), FBS (10%, PAN Biotech, #P30-3302), penicillin (100 U/ml) and streptomycin (0,1 mg/ml, Sigma-Aldrich, #P4333) at 37°C and 5% CO_2 in a humidified atmosphere. Triplicates 1 x 10^6 cells each were seeded in a 6 cm culture dish for 24h. After that, the cells were incubated overnight with basal medium, which differed in the absence of FBS. The cells were then treated with 1 µM **TFSM 1** or **TFSM 2** or the vehicle DMSO (Carl Roth, #4720.1) at 37°C for 24h. Finally, the medium was removed, the cells were washed twice with cold PBS (Sigma-Aldrich, #D8537), snap-frozen with liquid nitrogen and stored at -80°C. On the day of extraction, the cells were scratched with 200 µl PBS and lysed by vortexing (10 seconds at highest level before and after sonication) and sonication (10 min in a cold ultrasonic bath).

130 µl of cell lysate were subjected to lipid extraction using 1.5 ml methanol (VWR, #83638.320)/chloroform (Sigma-Aldrich, #1.02444, 2:1, v:v) as described..[5] The extraction solvent contained C17 ceramide (#860517), d_{31}-C16 sphingomyelin (#868584) and d_7-sphingosine (#860657, all Avanti Polar Lipids) as internal standards. Chromatographic separations were achieved on a 1290 Infinity II HPLC (Agilent Technologies) equipped with a Poroshell 120 EC-C8 column (3.0 x 150 mm, 2.7 µm; Agilent Technologies). MS/MS analyses were carried out using a 6495C triple-quadrupole mass spectrometer (Agilent Technologies) operating in the positive electrospray ionization mode (ESI+). HPLC conditions and ion source settings of the MS/MS detector have been described elsewhere.[6] TFSMs and their metabolites were analyzed by multiple reaction monitoring (MRM) using the following mass transitions (qualifier product ions in parentheses): sphingomyelin (SM) species: *m/z* 643.4 → 208.0 (125.0) for both **TFSM 1** and **TFSM 2**, and *m/z* 734.6 → 184.1 (86.1) for d_{31}-16:0 SM; ceramide (Cer) species: *m/z* 436.4 → 305.3 (323.3) for **TFSM 1**, *m/z* 436.4 → 264.3 (282.3) for **TFSM 2,** and *m/z* 534.5 → 264.3 (282.3) for C17 Cer; sphingosine (Sph) species: *m/z* 341.3 → 323.3 (293.3) for **TFSM 1,** *m/z* 300.3 → 282.3 (252.3) for **TFSM 2**, and *m/z* 307.3 → 289.3 (259.3) for d_7-Sph. Peak areas of SM, Cer and Sph species, as determined with MassHunter Quantitative Analysis software (version 10.1, Agilent Technologies), were normalized to those of the internal standards, d_{31}-16:0 SM, C17 Cer and d_7-Sph, respectively.

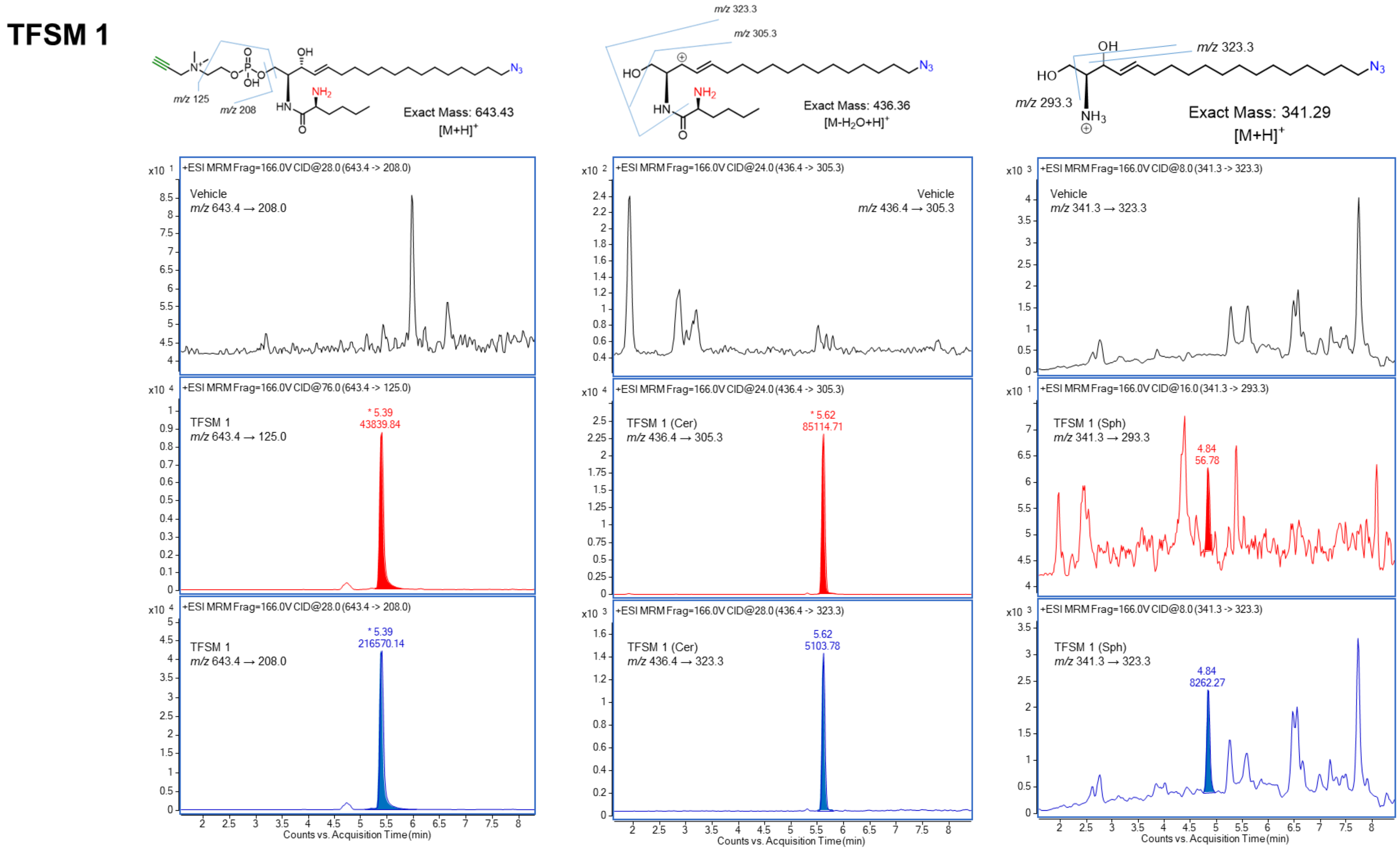

Supplementary Figure 2 Confirmation of cellular uptake and metabolization of TFSM 1 by LC-MS/MS. HEK293T cells were incubated with 1 µM **TFSM 1** for 24h. The cellular sphingolipids were then extracted and analyzed by LC-MS/MS. Shown are multiple reaction monitoring (MRM) chromatograms of **TFSM 1** (left panel), the ceramide (Cer) metabolite of **TFSM 1** after cleavage of the phosphocholine group (middle panel) and the sphingosine (Sph) derivative produced from **TFSM 1** (right panel). The chemical structures of the precursor ions and the proposed fragmentation sites are indicated at the top of the chromatograms. Each metabolite was identified by two characteristic mass transitions, shown in red and blue color, respectively. As a negative control, the quantifier mass transition (fragmentation with the highest signal intensity) of the solvent control (DMSO) is shown in black. Note that the y-axis of each chromatogram is scaled individually. Analyte peaks are labeled with retention time and peak area.

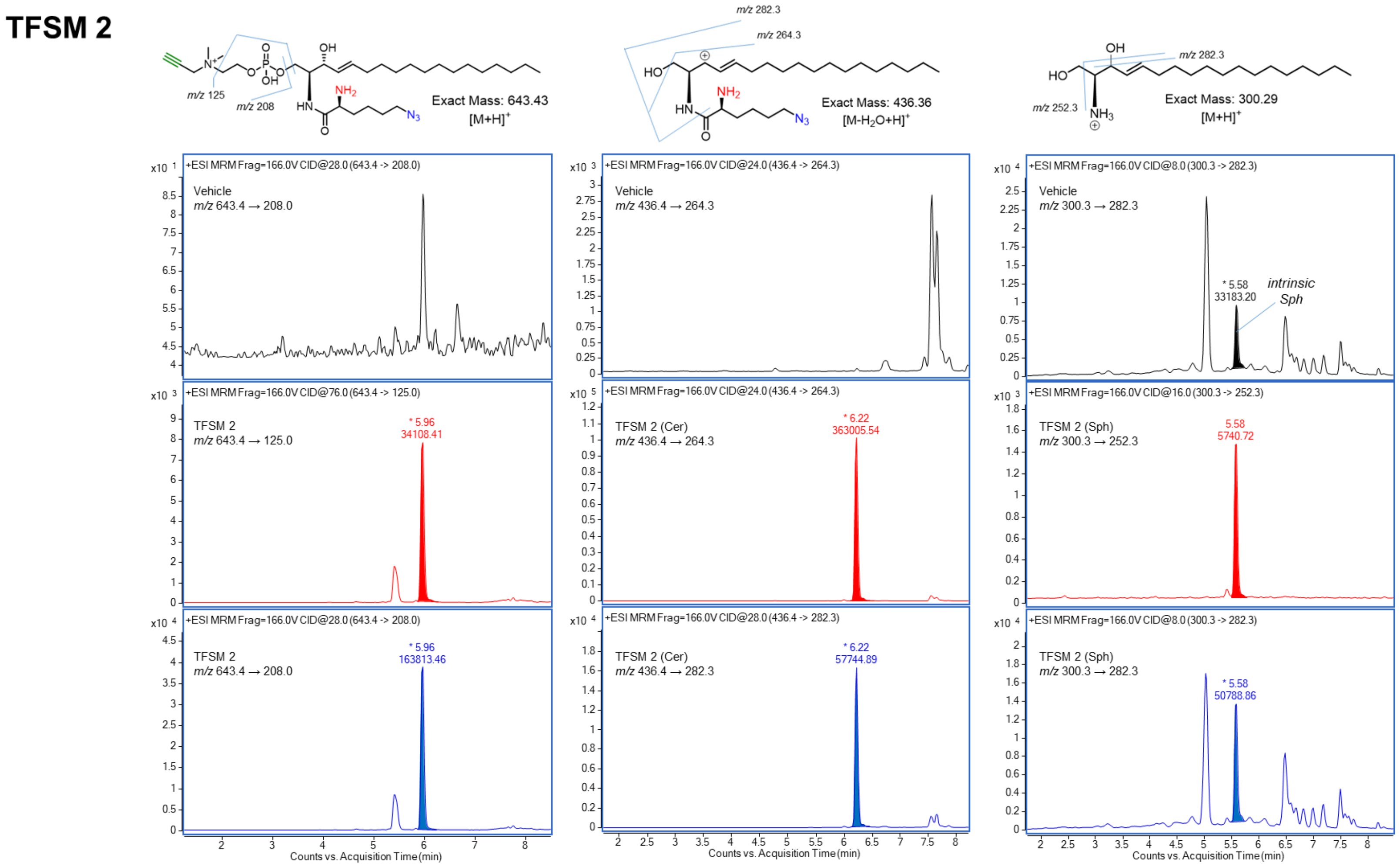

Supplementary Figure 3 Confirmation of cellular uptake and metabolization of TFSM 2 by LC-MS/MS. HEK293T cells were incubated with 1 µM **TFSM 2** for 24h. The cellular sphingolipids were then extracted and analyzed by LC-MS/MS. Shown are multiple reaction monitoring (MRM) chromatograms of **TFSM 2** (left panel), the ceramide (Cer) metabolite of **TFSM 2** after cleavage of the phosphocholine group (middle panel) and sphingosine (Sph), which is produced by **TFSM 2** or is intrinsically present in the cells (right panel). The chemical structures of the precursor ions and the proposed fragmentation sites are indicated at the top of the chromatograms. Each metabolite was identified by two characteristic mass transitions, shown in red and blue color, respectively. As a negative control, the quantifier mass transition (fragmentation with the highest signal intensity) of the solvent control (DMSO) is shown in black. Note that the y-axis of each chromatogram is scaled individually. Analyte peaks are labeled with retention time and peak area

Supplementary Figures

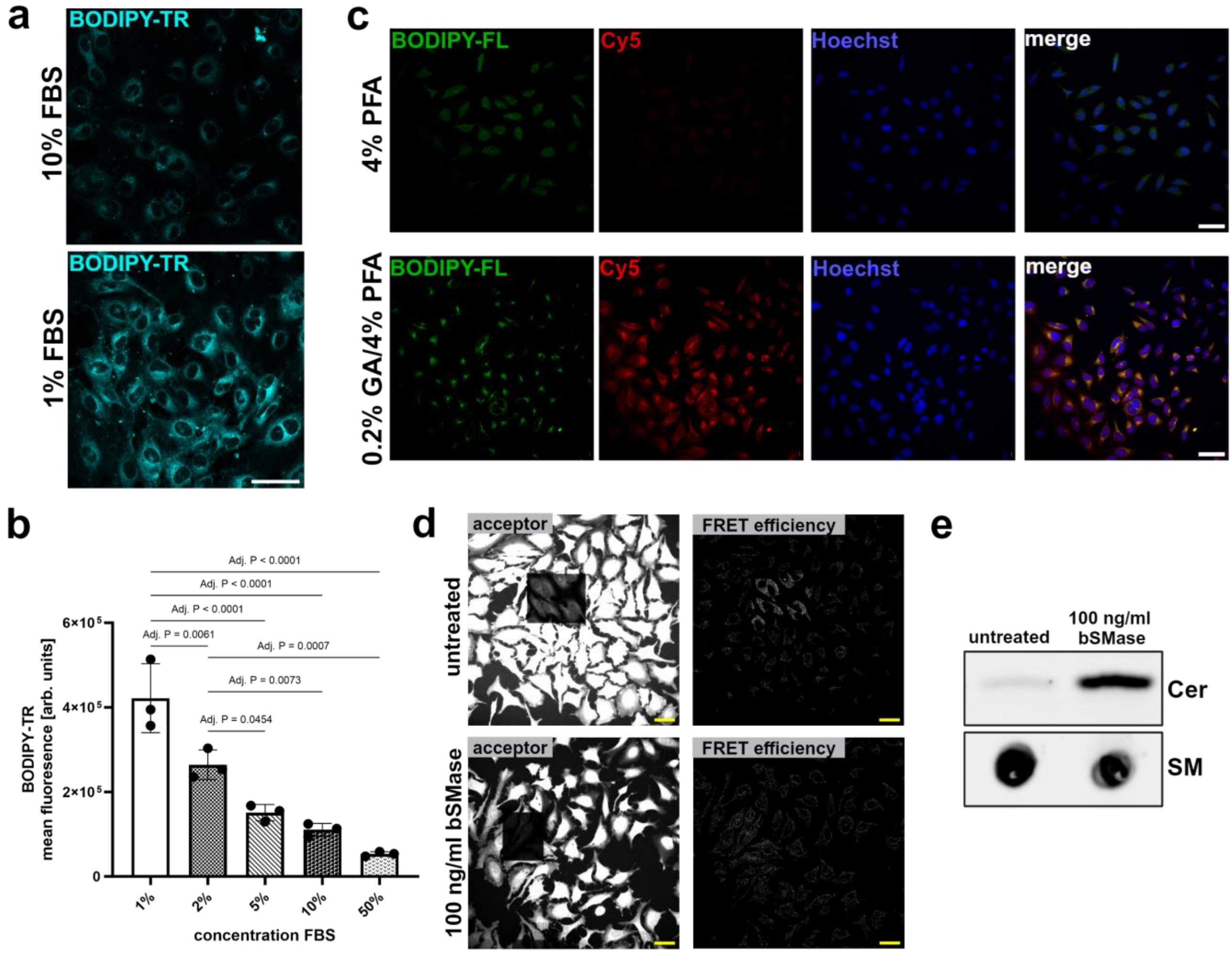

Supplementary Figure 4 Sample preparation and SMase activity measurements with TFSM derivatives
a and **b**, cellular uptake of the visible-range FRET probe is impaired by high FBS concentrations. HeLa cells were treated for 2h with 10 µM visible range FRET probe in presence of 1% or 10% FBS. Then, samples were either fixed and analyzed with Leica TCS SP5 microscope (a, n=1) or cells were detached, and BODIPY-TR fluorescence was determined with a Attune^{TM} NxT flow cytometer (b, n=3). Statistics: One-way ANOVA and Tukey's multiple comparisons. Scale bars: 50 µm. **c**, Fixation of TFSMs requires glutaraldehyde. HeLa cells were incubated with **TFSM 1** in presence of 1% FBS for 2h and either fixed with 4%paraformaldehyde (PFA) or a mixture 4% PFA and 0.2% glutaraldehyde (GA). **TFSM 1** was stained with BODIPY-FL-DBCO or Cy5 azide and Nuclei were stained with Hoechst. Images recorded with Leica TCS SP5 microscope. n=1 Scale bars: 50 µm. **d**, Metabolization of **TFSM 1** by β-toxin can be monitored via FRET. HeLa cells were incubated with 10 µM **TFSM 1** for 2h. Then, the compound was removed, and cells were either treated with 100 ng/ml β-toxin or left untreated for 3h. Then, samples were fixed and clicked with BODIPY-FL-DBCO (backbone) and AlexaFluor^{TM}546 (headgroup). FRET efficiency was determined by acceptor bleaching with a confocal Leica TCS SP5 microscope and the built-in FRET AB wizard. n=3, Scale bars: 25 µm. **e**, bSMase treatment reduces cellular levels of BODIPY-FL-C_{12}-SM. HeLa cells were incubated with BODIPY-FL-C_{12}-SM, the compound was removed, and cells were treated with 100 ng/ml bSMase. Cells were detached and lipids were extracted by 2:1 $CHCl_3$:MeOH. BODIPY-FL-C_{12}-ceramide and BODIPY-FL-C_{12}-SM were separated by thin layer chromatography and scanned with a Typhoon RGB Scanner. n=3. Bars represent means ± SD. n corresponds to biological replicates. Source data and detailed statistics are provided as a Source Data file.

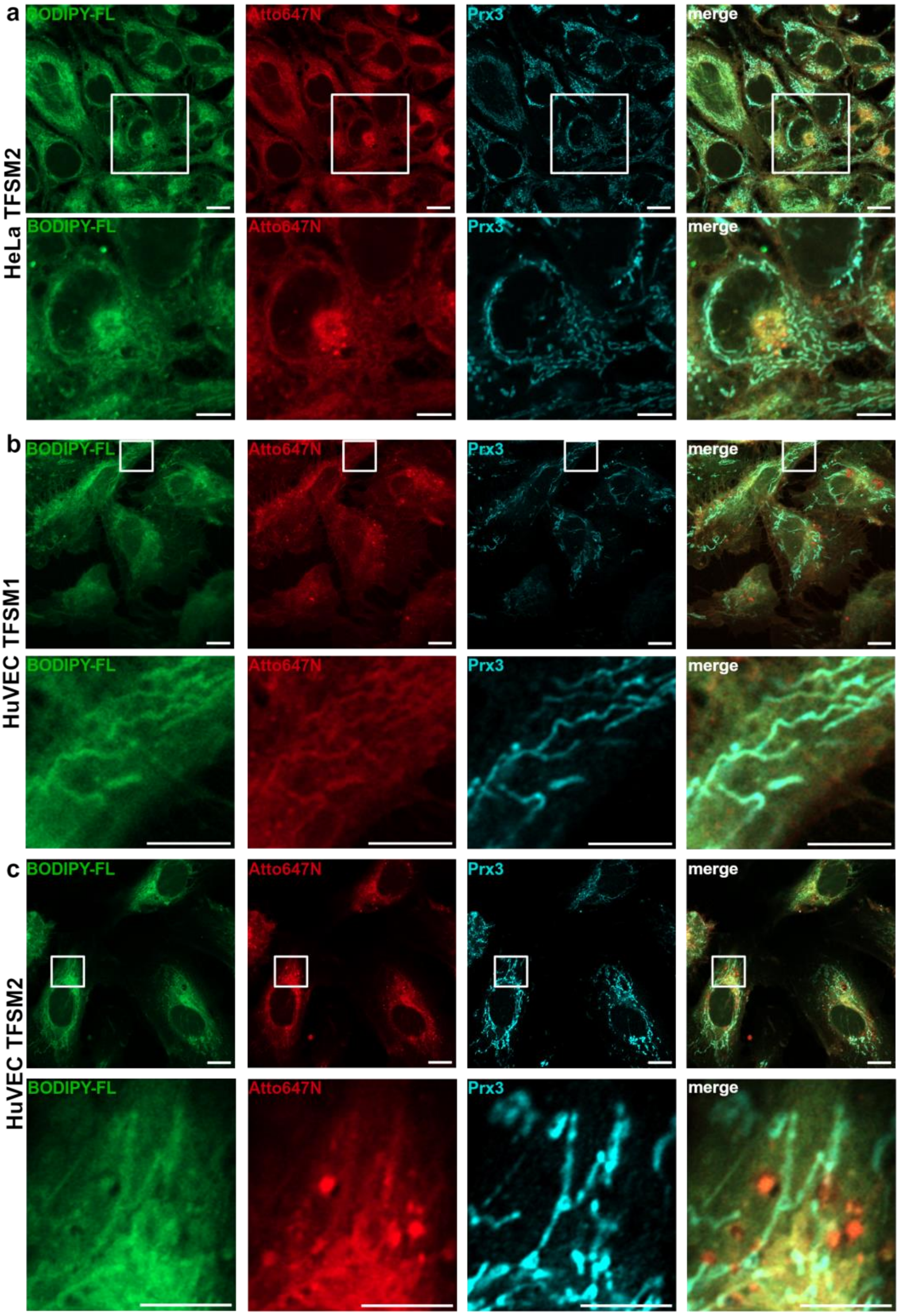

Supplementary Figure 5 TFSMs are incorporated to mitochondria
HeLa (a) or HuVEC (b , c) were incubated either with TFSM1 (b) or TFSM2 (a, c) for 24h. Samples were fixed, and mitochondria were stained with a Prx3 primary and a CF568-conjugated secondary antibody. The TFSM backbone was stained with BODIPY-FL-DBCO and the headgroup was stained with Atto647N azide. Imaging was performed with a Zeiss LSM900 microscope equipped with Airyscan 2. n=1 Scale bars: 10 µm, zoomed images 5 µm.

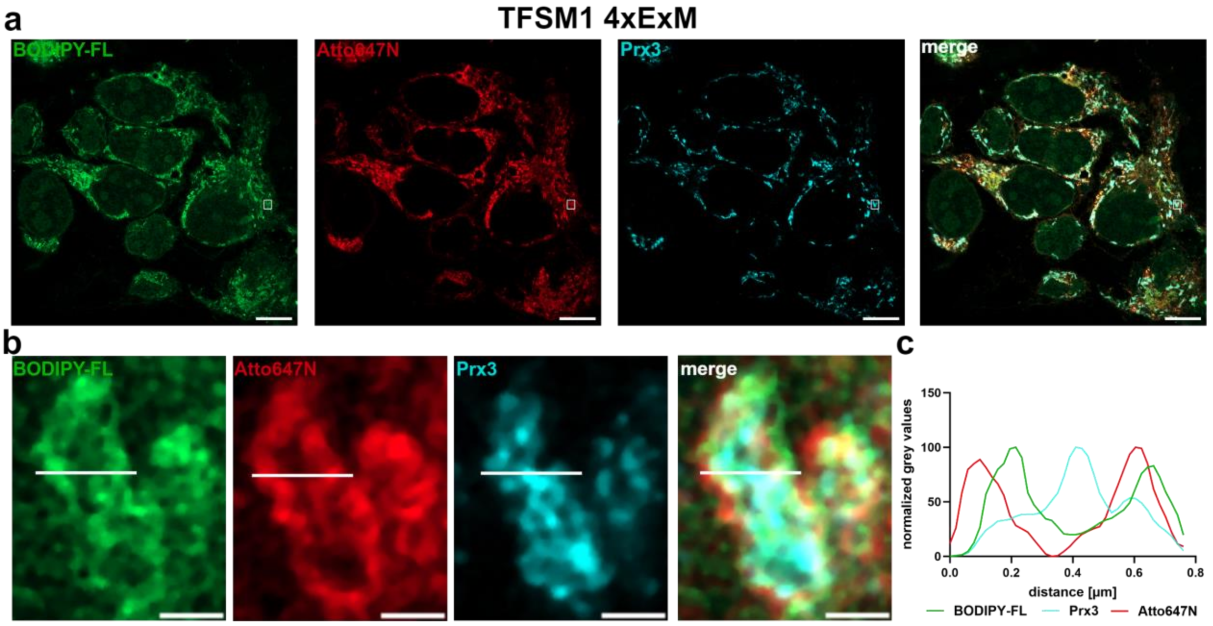

Supplementary Figure 6 ExM of TFSMs enables visualization of mitochondrial envelope.
a, HeLa cells were incubated with TFSM1 for 24h. Samples were fixed, and mitochondria were stained with a Prx3 primary and a CF568-conjugated secondary antibody. The TFSM backbone was stained with BODIPY-FL-DBCO and the headgroup was stained with Atto647N azide. Samples were 4-fold expanded. Imaging was performed with a Zeiss LSM900 microscope equipped with Airyscan 2. n=1 Scale bars: 40 µm (with 4-fold expansion factor ~10 µm), **b,** Zoomed images from a, Scale bars: 2 µm (with 4-fold expansion factor ~0.5 µm).**c**, Signal intensity was measured in Fiji (ROI indicated by white line in b) and results were normalized to the highest and lowest grey value detected in the respective channel. n=1.

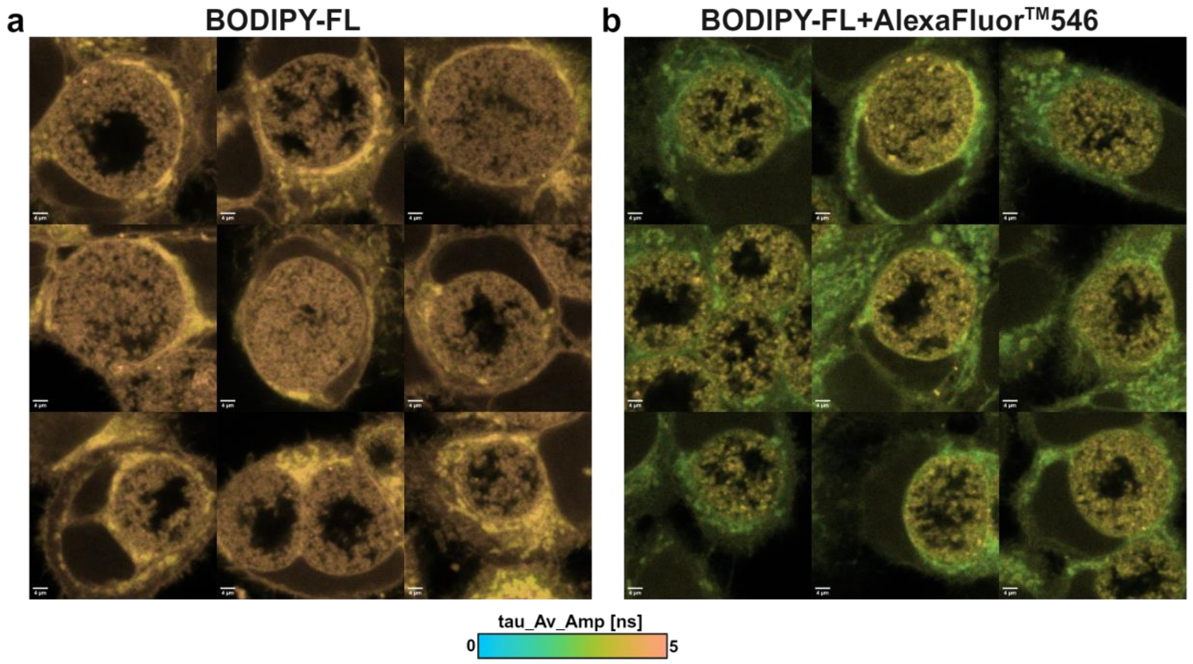

Supplementary Figure 7 FLIM FRET analysis of Chlamydia-infected cells with TFSM1
FLIM images of infected, TFSM1-stained, HeLa cells click-labeled with BODIPY-FL-DBCO (a) as well as BODIPY-FL-DBCO and AlexaFluor^{TM} 546-azide (b) measured by single-photon sensitive confocal fluorescence lifetime microscopy excited at 488 nm with 0.5 kW cm-2 at an integration time of 25 µs pixel^{-1}. The samples were measured 9 - 10 times independently and no intensity threshold was applied. n=1 Scale bars: 4 µm.

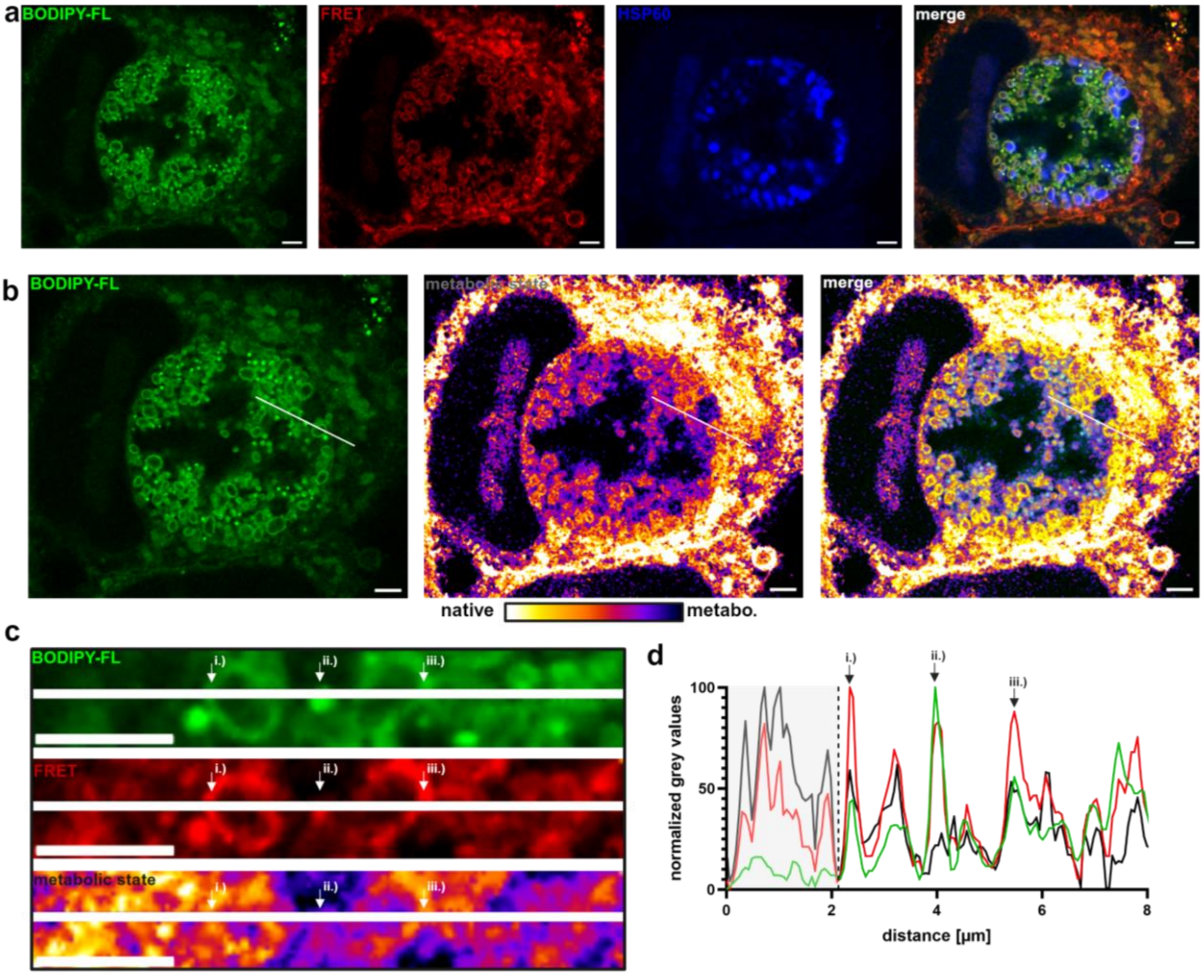

Supplementary Figure 8 Elementary bodies contain a higher proportion of metabolized TFSM2 than reticulate bodies. a, TFSM can be used to visualize chlamydial inclusion via 4x ExM. HeLa cells were infected with C. trachomatis at MOI1 in presence of **TFSM 1** for 24h, fixed and stained with AlexaFluor^{TM}546-azide (headgroup) and BODIPY-FL-DBCO (backbone). *Chlamydia* were visualized with a anti-chlamydial HSP60 primary and a AlexaFluor^{TM}405-conjugated secondary antibody. Samples were 4x expanded and imaged with a Leica TCS SP5 microscope. Scale bars: 8 µm (with 4-fold expansion factor ~2 µm). **b**, Periphery of inclusions predominantly contain non-metabolized TFSM compared to their center. The image depicted in a.) was zoomed and the metabolic state of TFSM was calculated by determining the ratio of FRET vs. donor (BODIPY-FL) signal. White arrows indicate intermediate chlamydial developmental forms. Scale bars: 8 µm (with 4-fold expansion factor ~2 µm). **c**, and **d**, Sphingolipid composition of EBs and RBs differ. The area indicated by a white line in b.) was magnified and the intensity profile was measured in Fiji. The resulting grey values were scaled to the highest and lowest value detected in individual channels. n=2. Scale bar 4 µm (with 4-fold expansion factor ~1 µm).

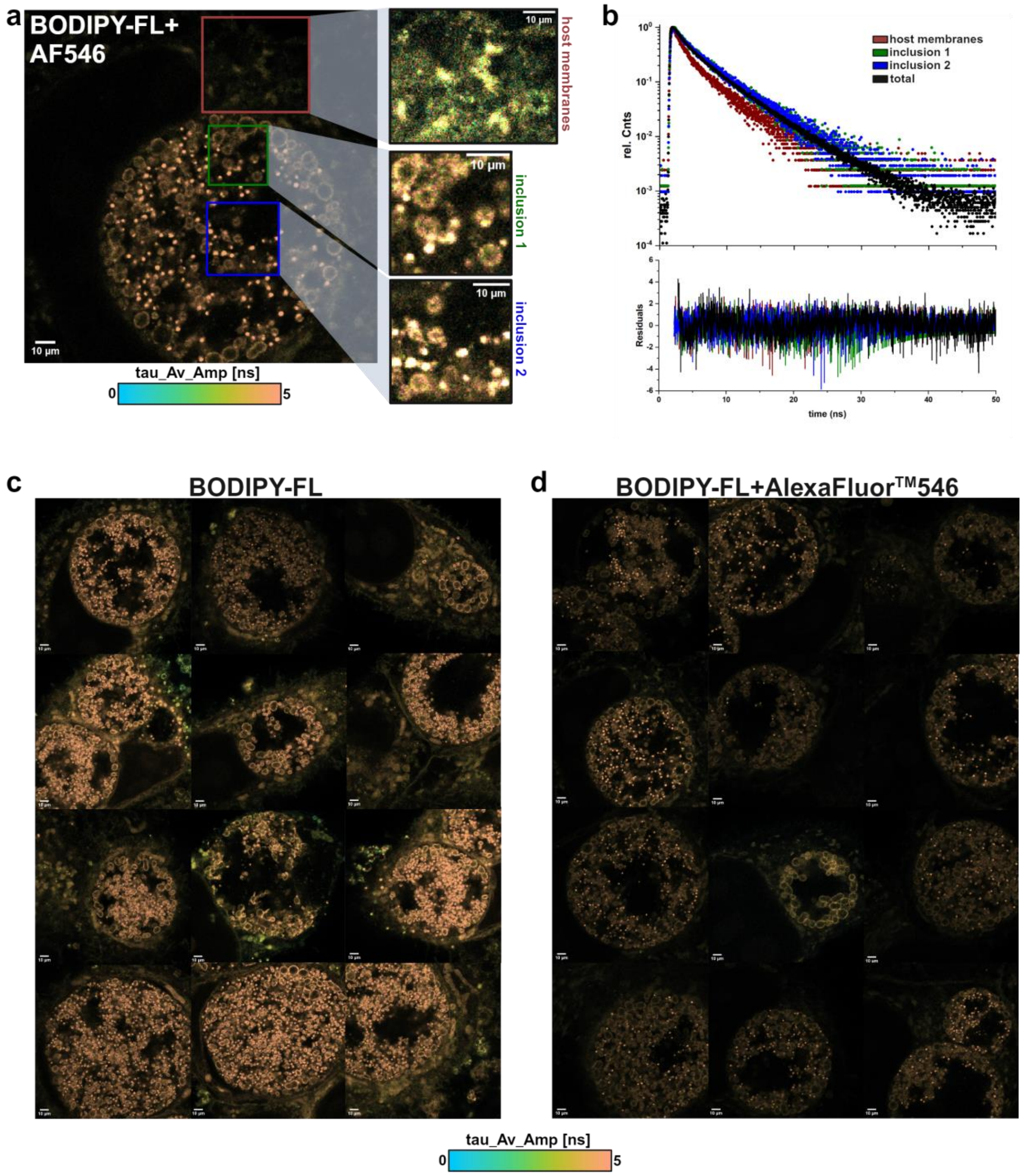

Supplementary Figure 9 FLIM FRET analysis of *Chlamydia*-infected cells with TFSM1 via ExM

a, FLIM image of a 4-fold expanded, TFSM1-stained, HeLa cell click-labeled with BODIPY-FL-DBCO and AlexaFluor^{TM} 546-azide. No intensity threshold was applied. The marked insets give a detailed view of the fluorescence lifetime distribution and show a reduced fluorescence donor lifetime at the host cell membrane (red) in comparison to the *Chlamydia* inclusion (green, blue) as well as a higher fluorescence lifetime of EBs in comparison to RBs. n=1. Scale bars: 10 µm. b, average fluorescence lifetime decay of the overall image (black decay) as well as of the marked insets showed in a. The color of the fluorescence lifetime decays corresponds to the colors of the insets in a. c and d, subset of FLIM images of 12 infected, TFSM1-stained, HeLa cells click-labeled with BODIPY-FL-DBCO (c) as well as BODIPY-FL-DBCO and AlexaFluor^{TM} 546-azide (d) measured by confocal fluorescence lifetime microscopy excited at 488 nm with ~7 kW cm-2 at an integration time of 25 µs pixel-1. The samples were measured 12 times independently and no intensity threshold was applied. n=1 Scale bars: 10 µm.

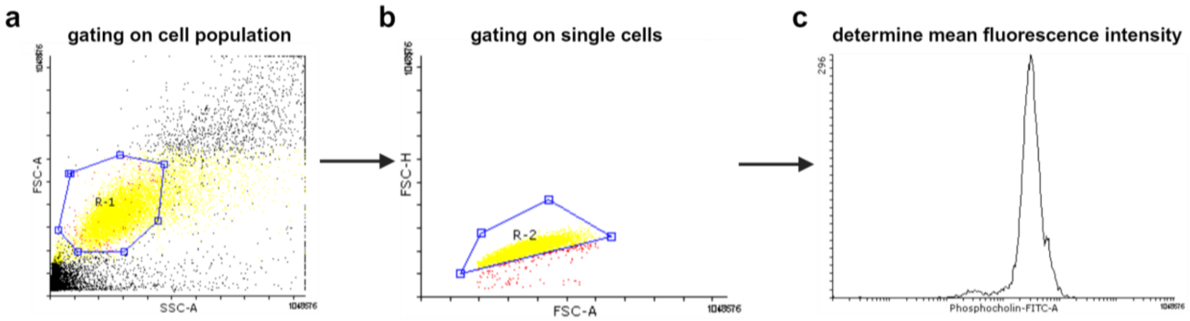

Supplementary Figure 10 Gating strategy to determine fluorescence mean intensity in a cell population. First, the cell population was selected by forward scatter peak area (FSC-A) and side scatter peak area (SSC-A, a). The resulting population was gated on single cells by FSC-A and forward scatter peak height (FSC-H, b). Single cells were analyzed for BODIPY-FL (FITC) mean fluorescence intensity (c).

NMR Spectra

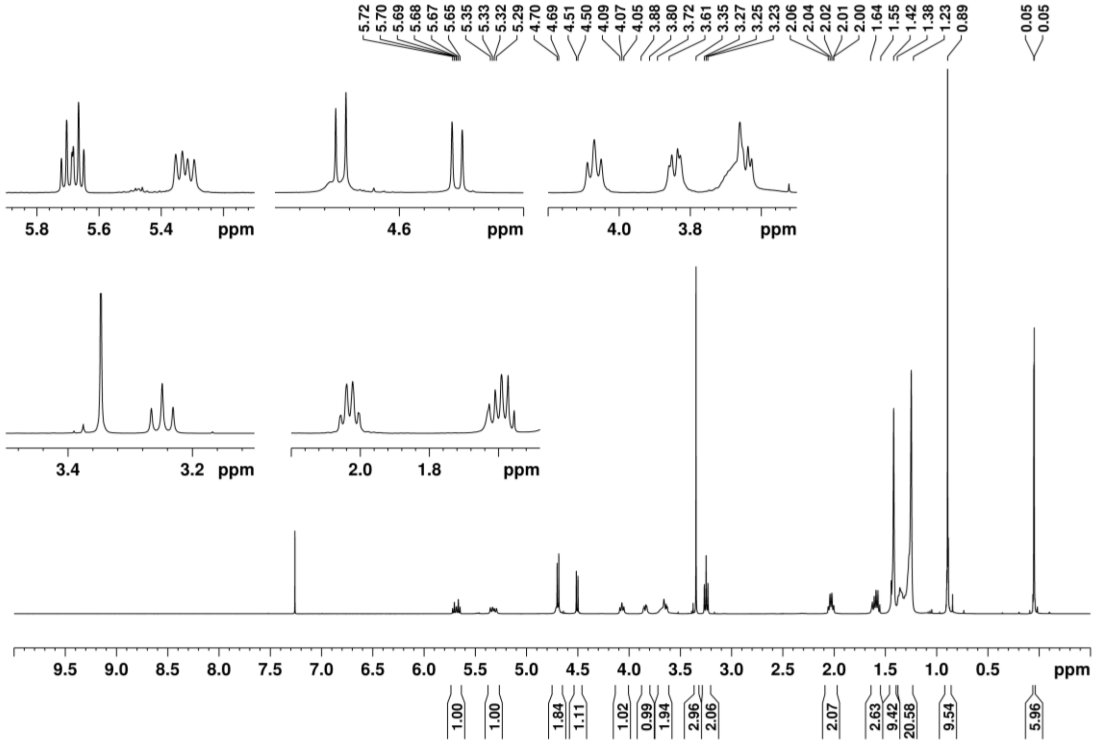

Supplementary Figure 11 ^{1}H NMR spectrum (400 MHz, $CDCl_3$) of compound 5.

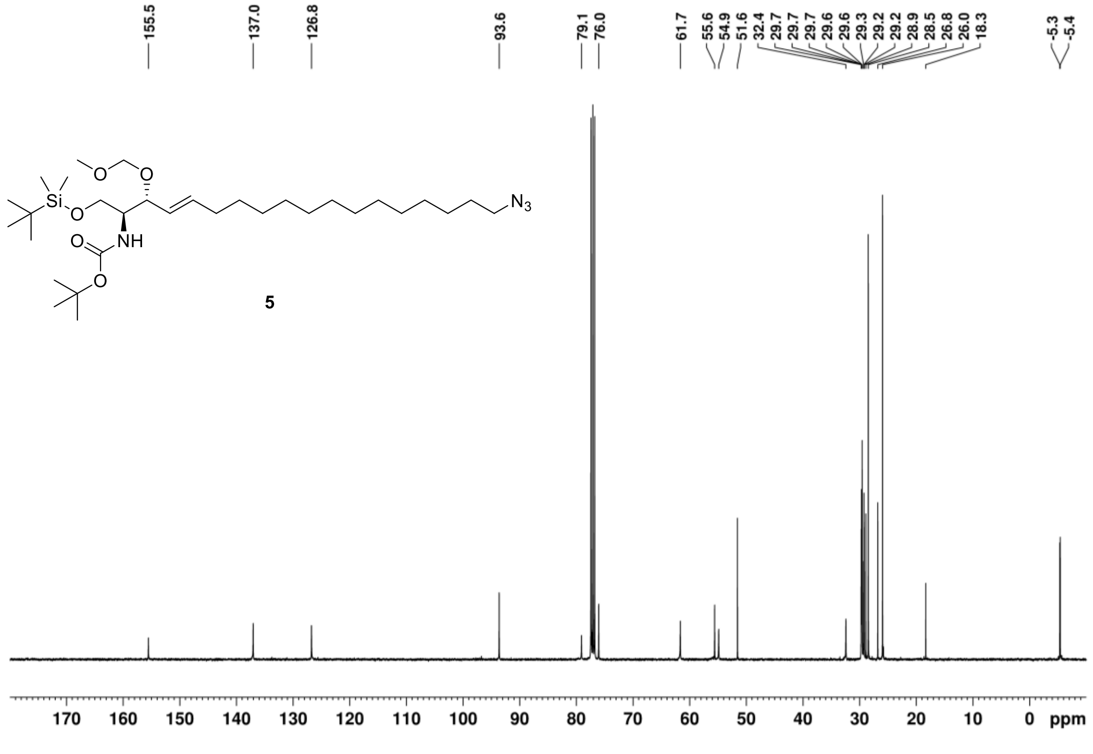

Supplementary Figure 12 ^{13}C NMR spectrum (100 MHz, $CDCl_3$) of compound 5.

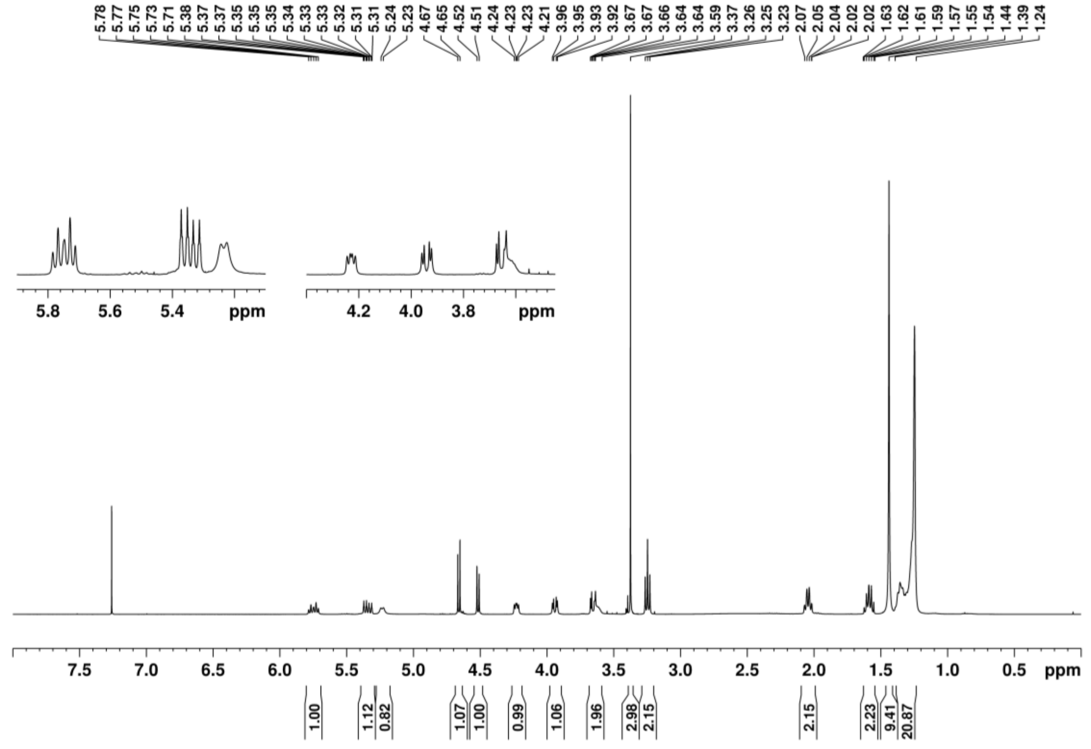

Supplementary Figure 13 ^{1}H NMR spectrum (400 MHz, $CDCl_3$) of compound 7.

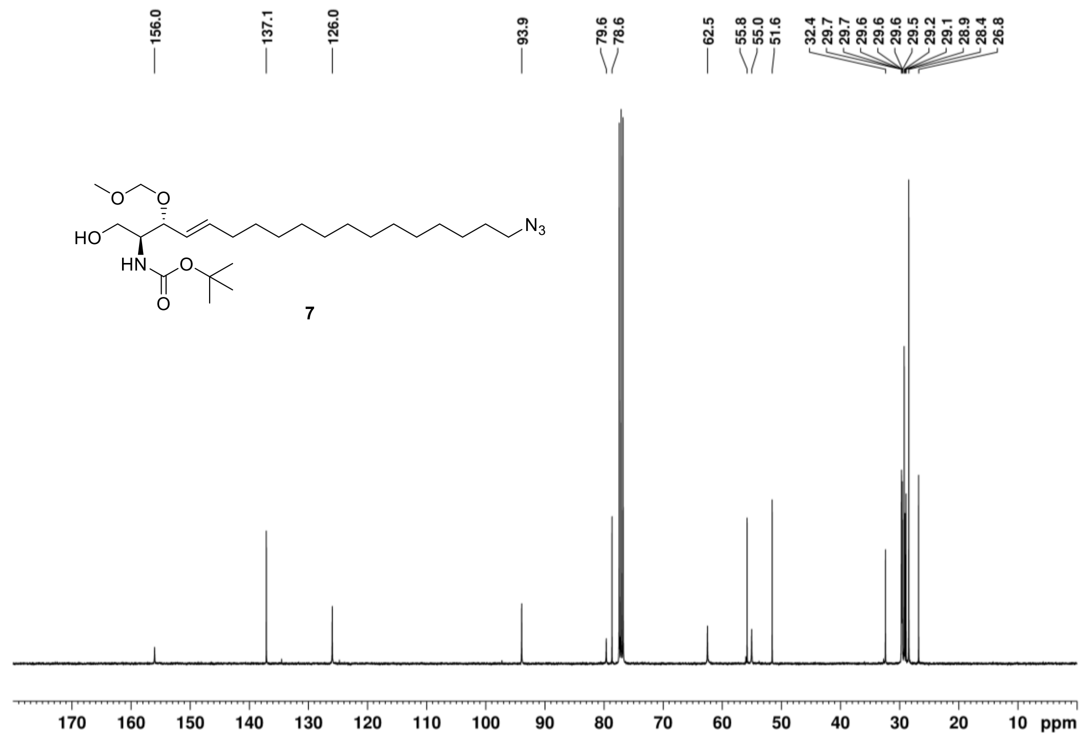

Supplementary Figure 14 ^{13}C NMR spectrum (100 MHz, $CDCl_3$) of compound 7.

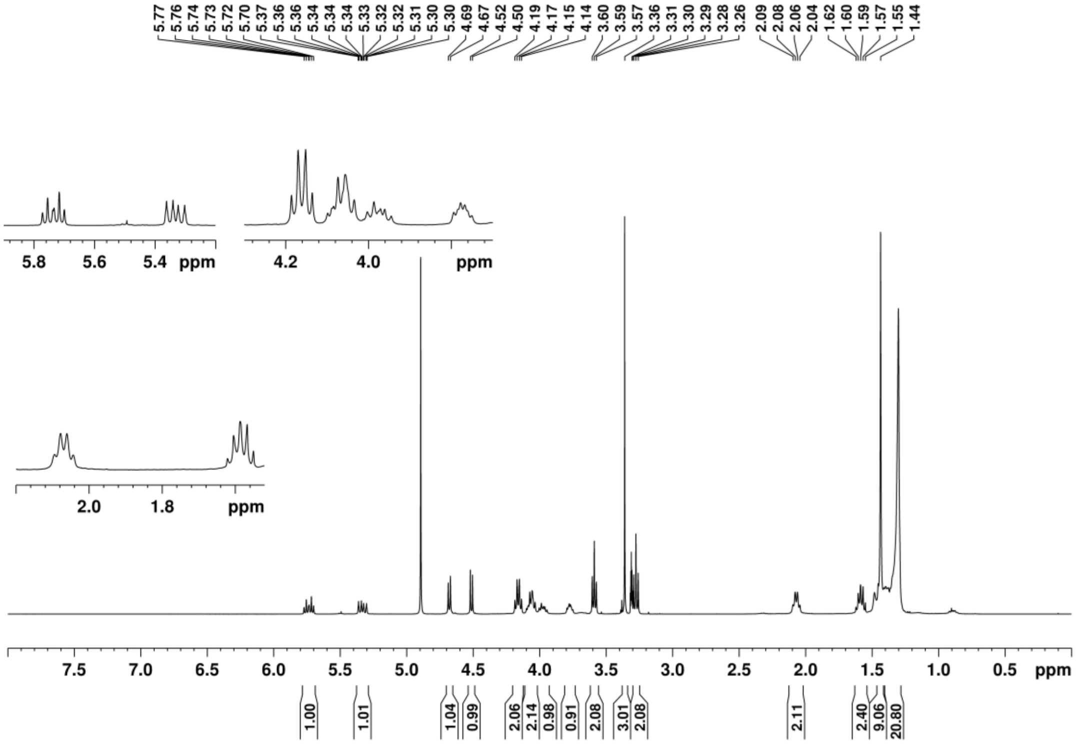

Supplementary Figure 15 ^{1}H NMR spectrum (400 MHz, CD_3OD) of compound 9.

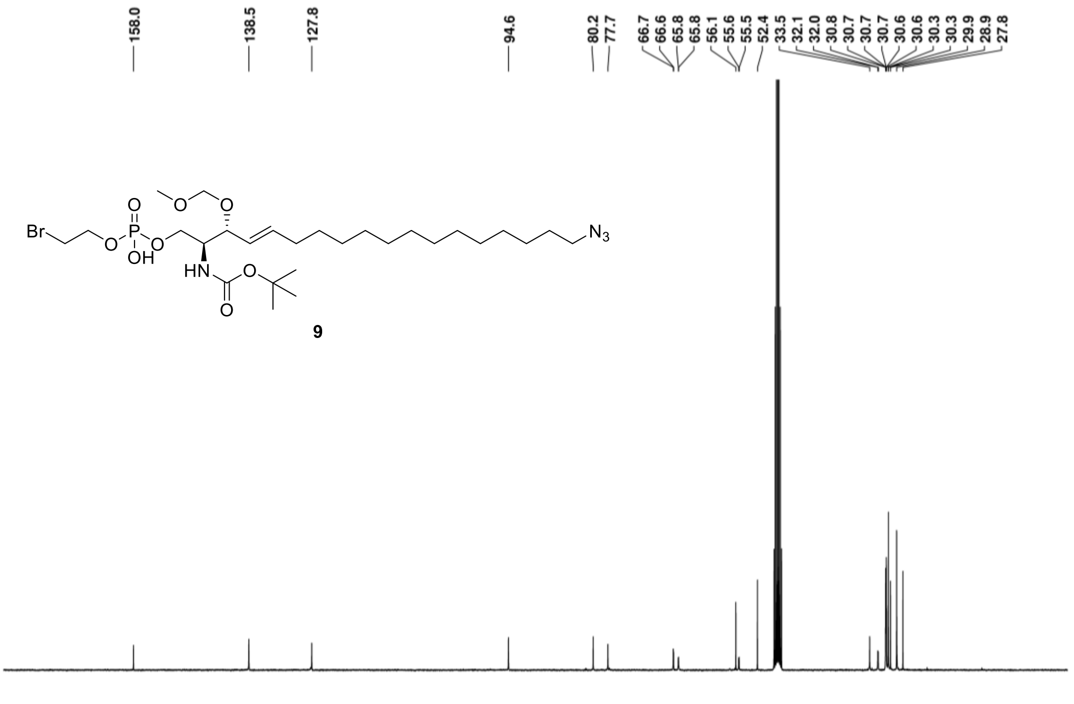

Supplementary Figure 16 ^{13}C NMR spectrum (100 MHz, CD_3OD) of compound 9.

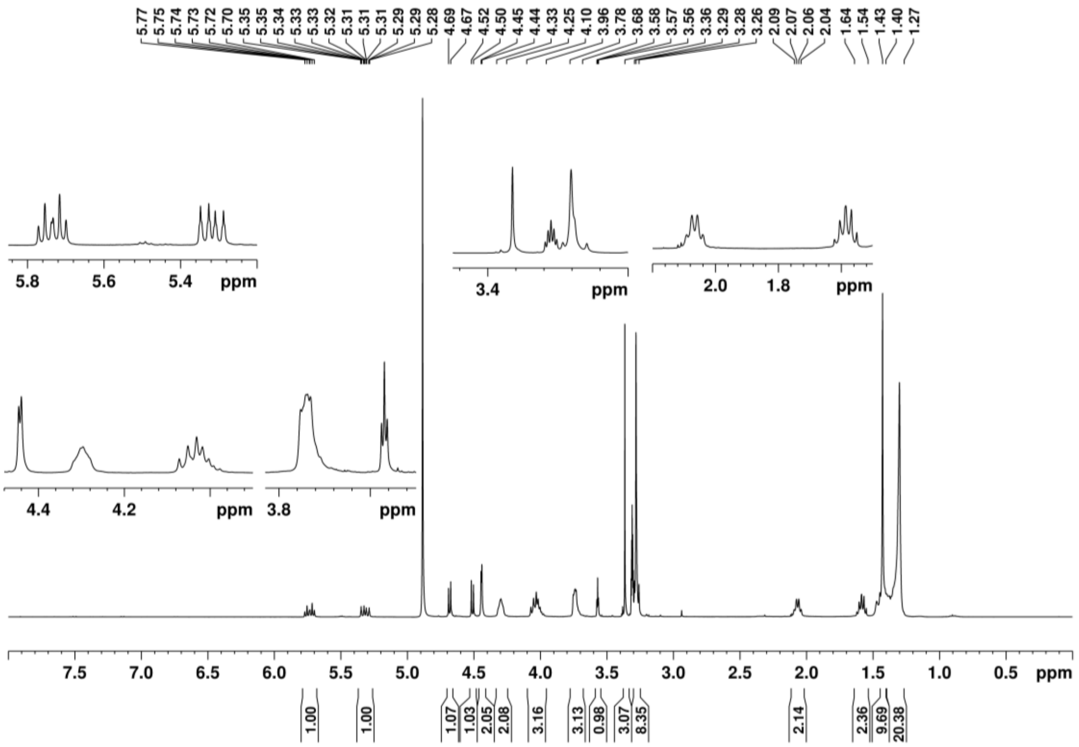

Supplementary Figure 17 ^{1}H NMR spectrum (400 MHz, CD_3OD) of compound 11.

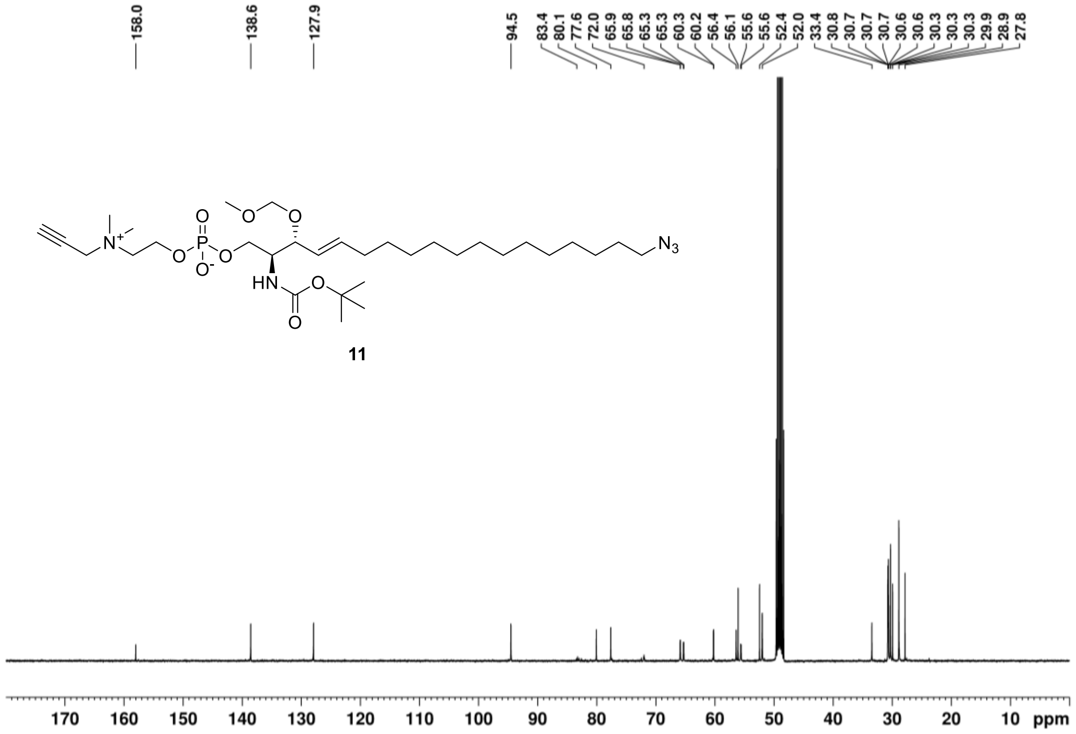

Supplementary Figure 18 ^{13}C NMR spectrum (100 MHz, CD_3OD) of compound 11.

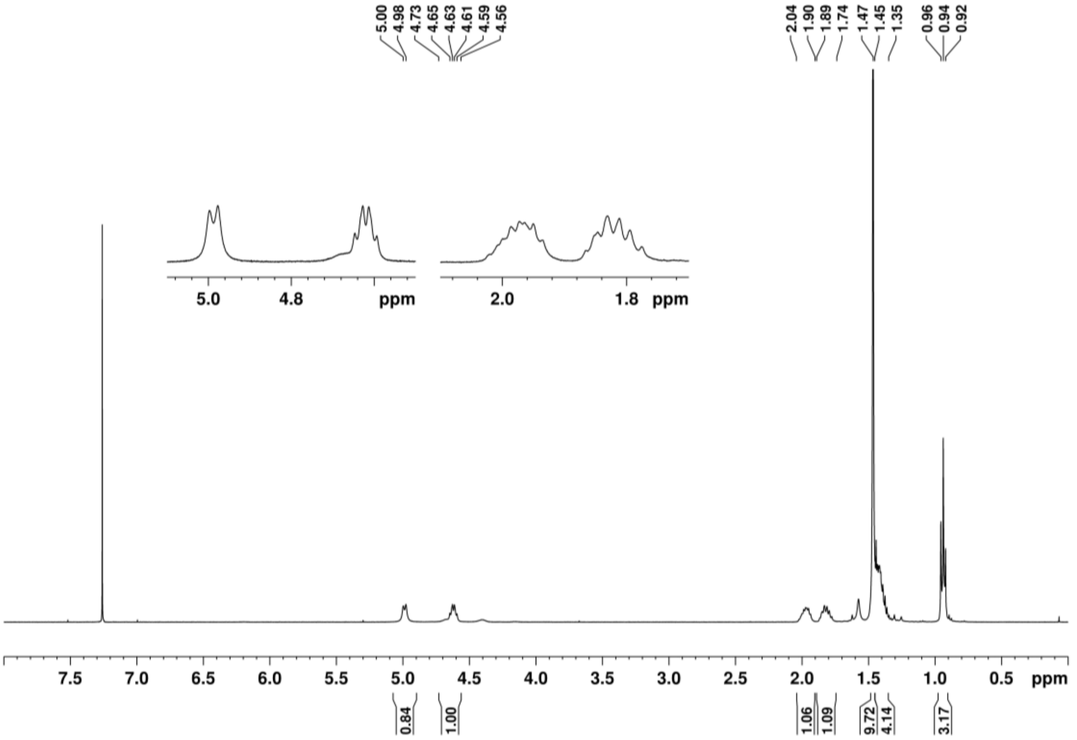

Supplementary Figure 19 ^{1}H NMR spectrum (400 MHz, $CDCl_3$) of compound 13.

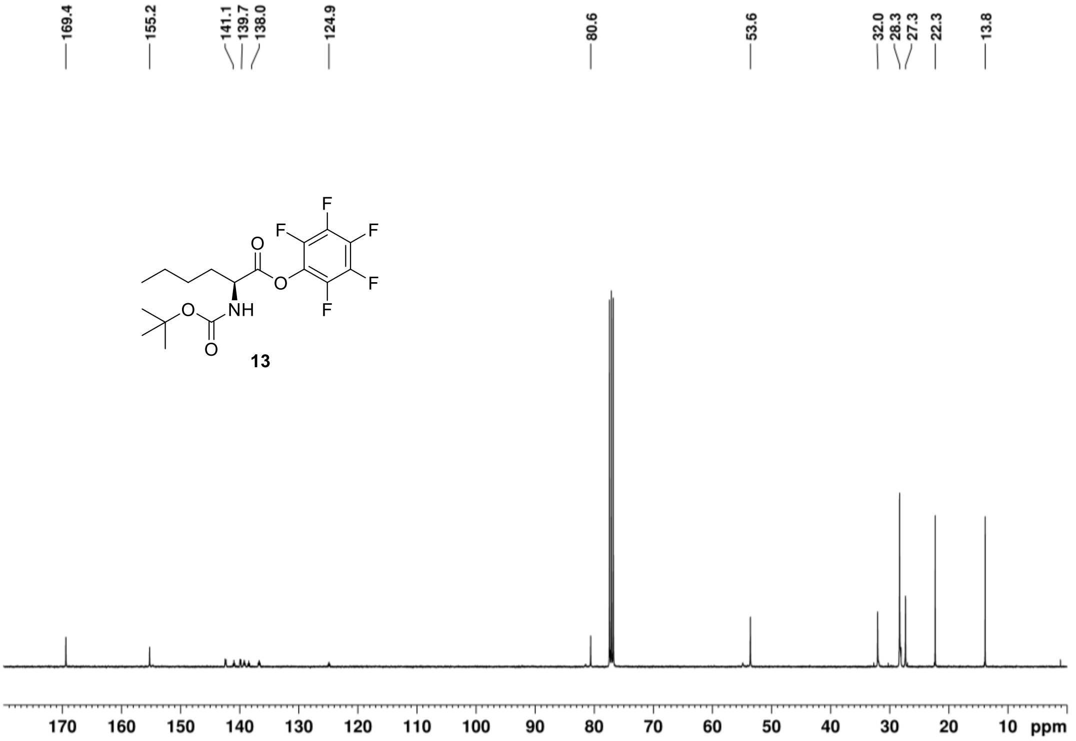

Supplementary Figure 20 ^{13}C NMR spectrum (100 MHz, $CDCl_3$) of compound 13.

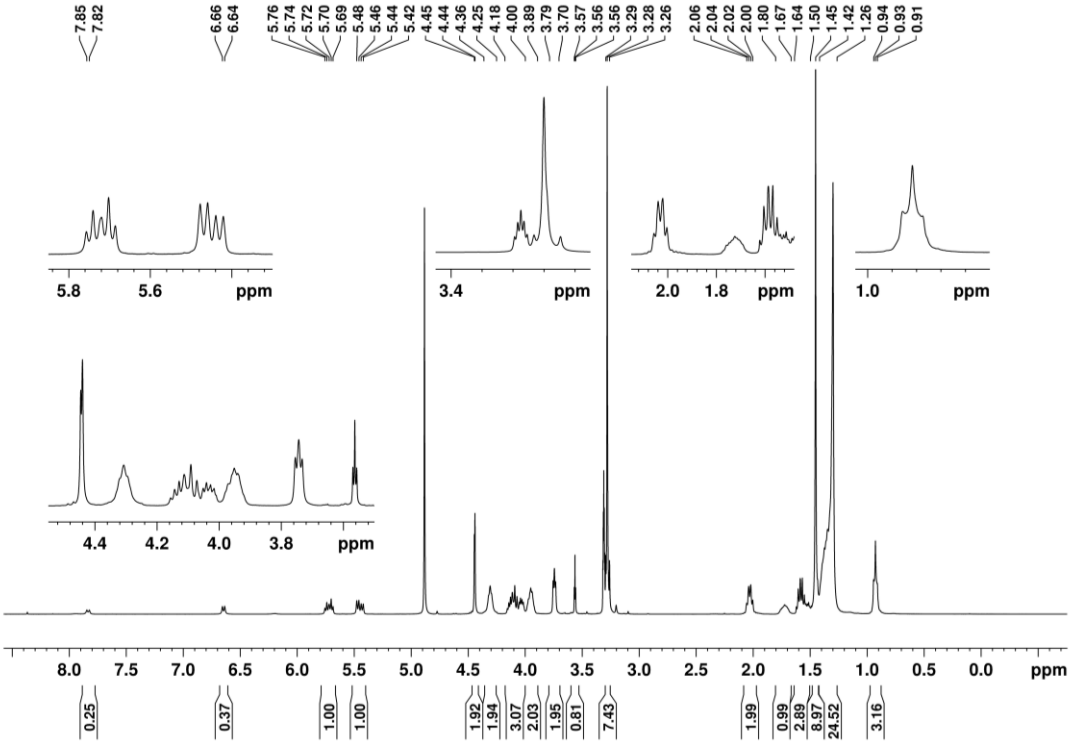

Supplementary Figure 21 ^{1}H NMR spectrum (400 MHz, CD_3OD) of compound 15.

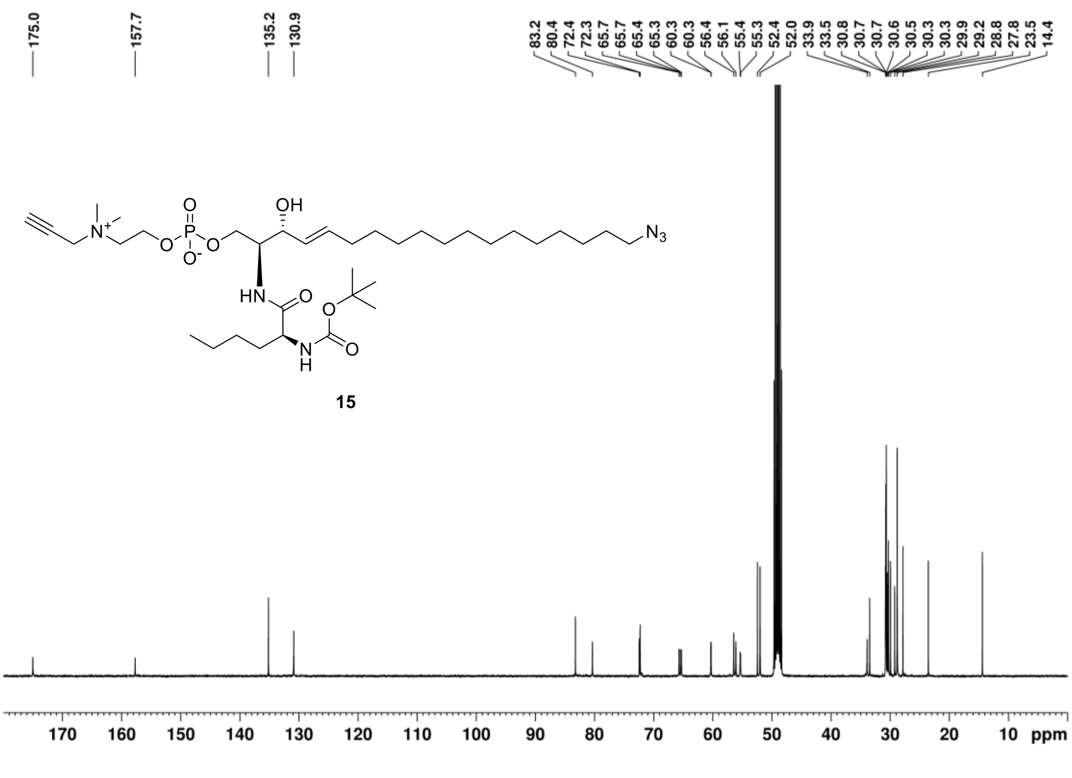

Supplementary Figure 22 ^{13}C NMR spectrum (100 MHz, CD_3OD) of compound 15.

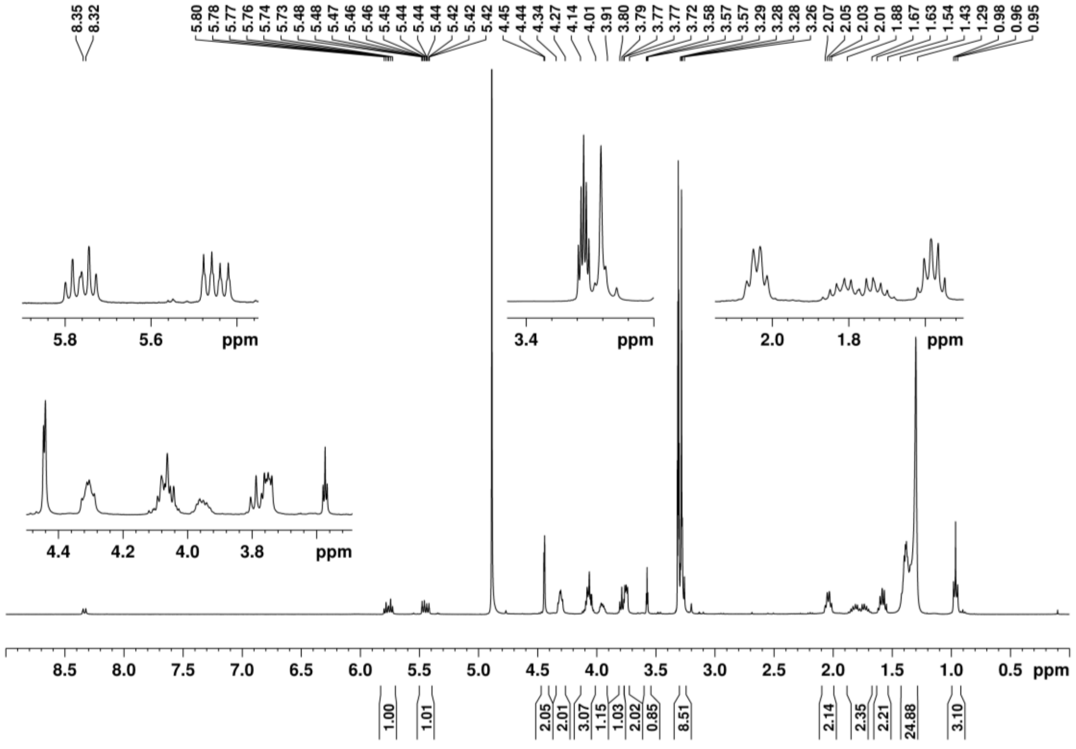

Supplementary Figure 23 ^{1}H NMR spectrum (400 MHz, CD_3OD) of target molecule TFSM 1.

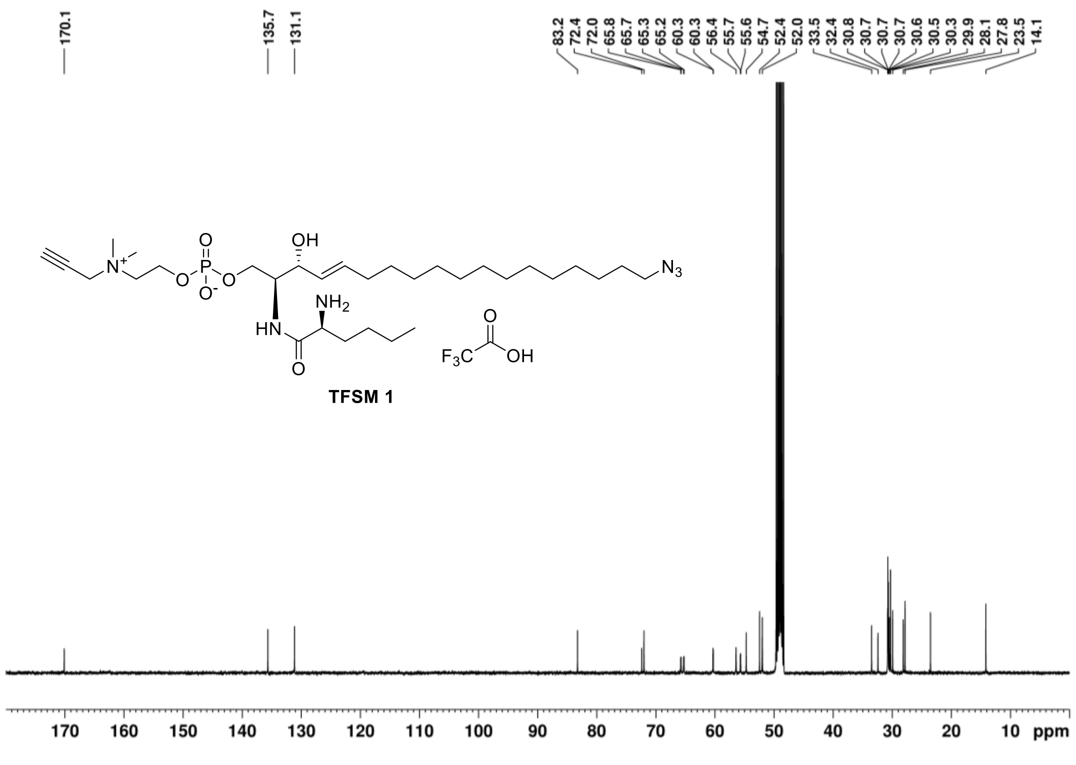

Supplementary Figure 24 ^{13}C NMR spectrum (100 MHz, CD_3OD) of target molecule TFSM 1.

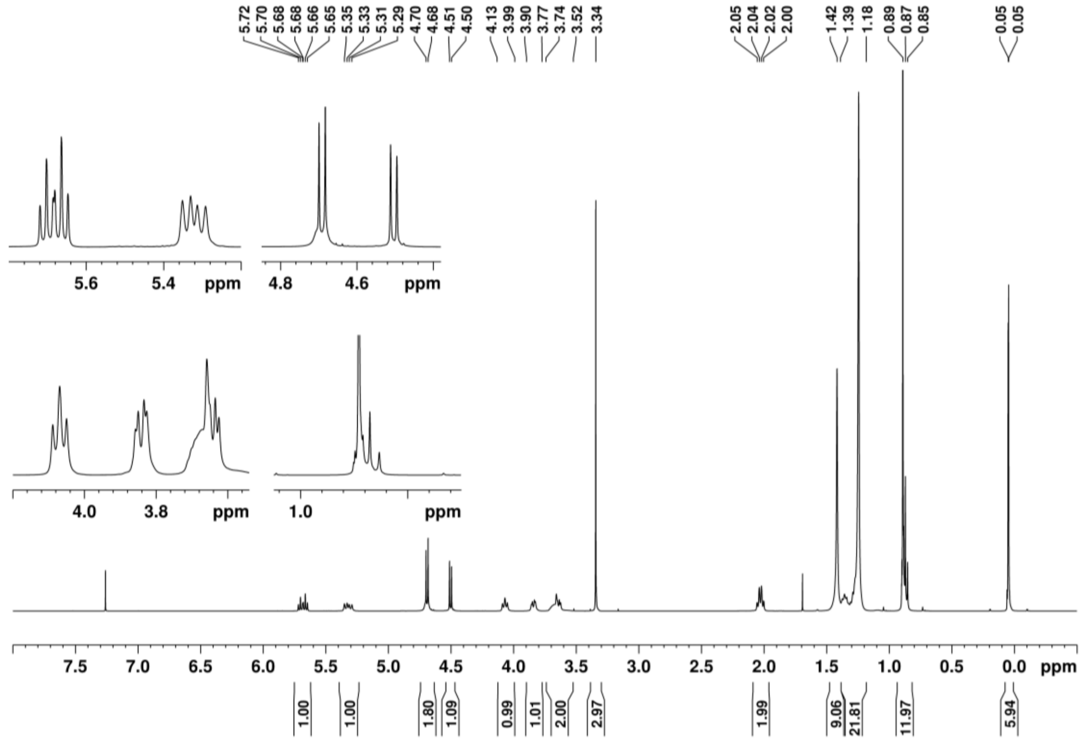

Supplementary Figure 25 ^{1}H NMR spectrum (400 MHz, $CDCl_3$) of compound 6.

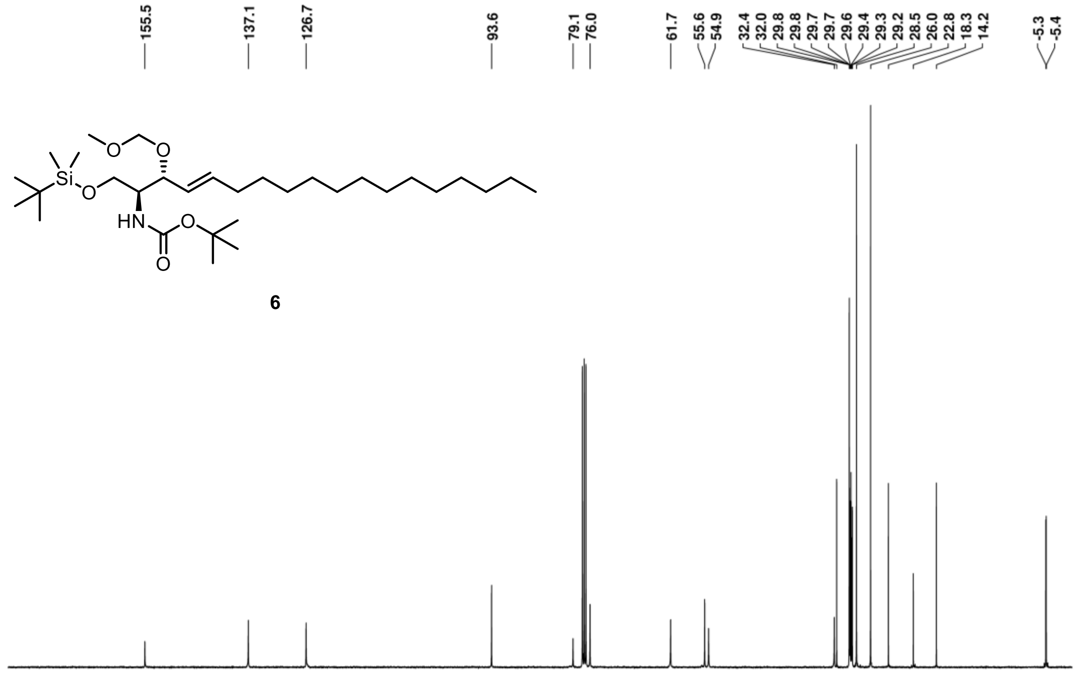

Supplementary Figure 26 ^{13}C NMR spectrum (100 MHz, $CDCl_3$) of compound 6.

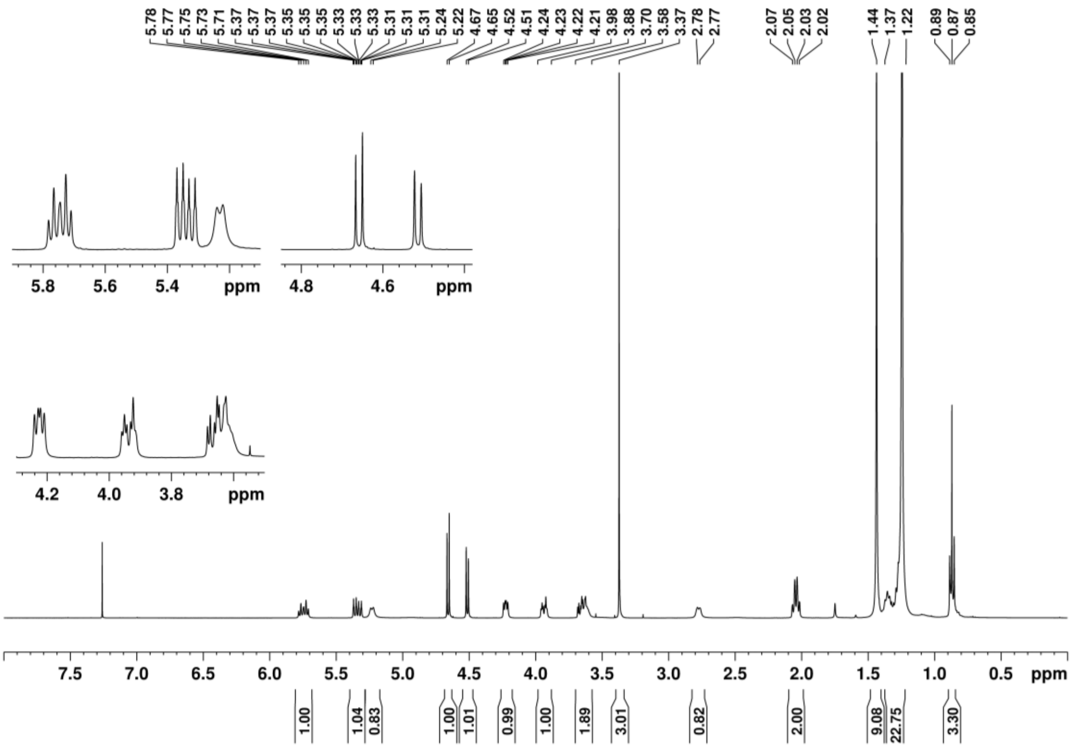

Supplementary Figure 27 ^{1}H NMR spectrum (400 MHz, $CDCl_3$) of compound 8.

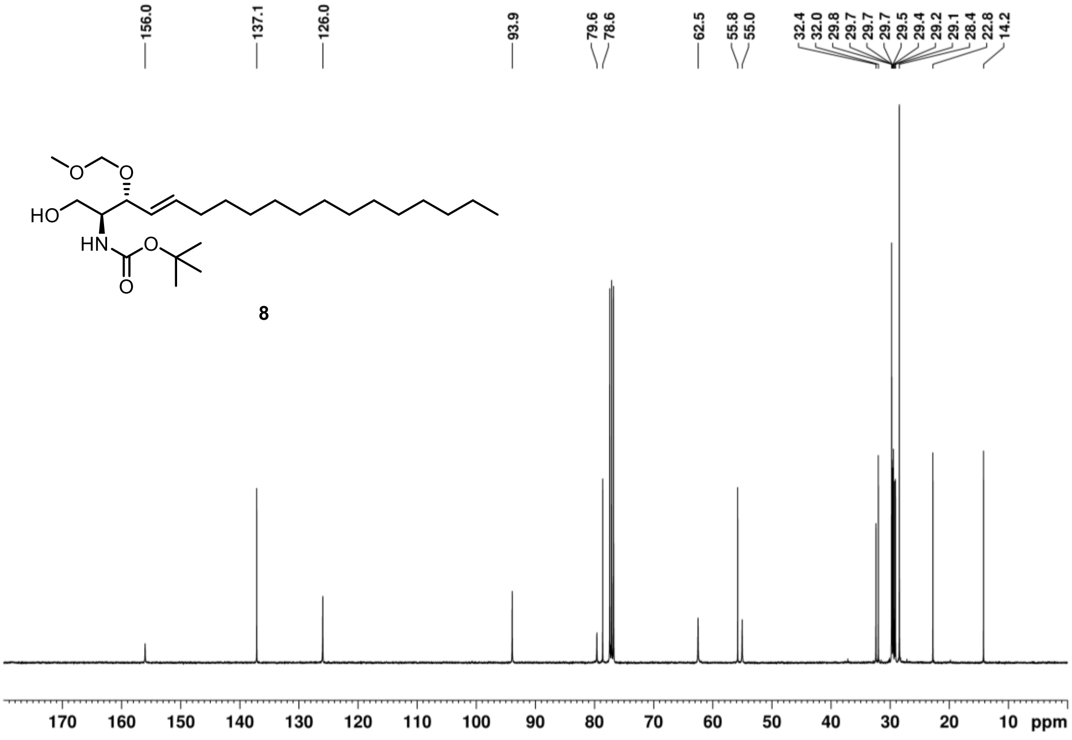

Supplementary Figure 28 ^{13}C NMR spectrum (100 MHz, $CDCl_3$) of compound 8.

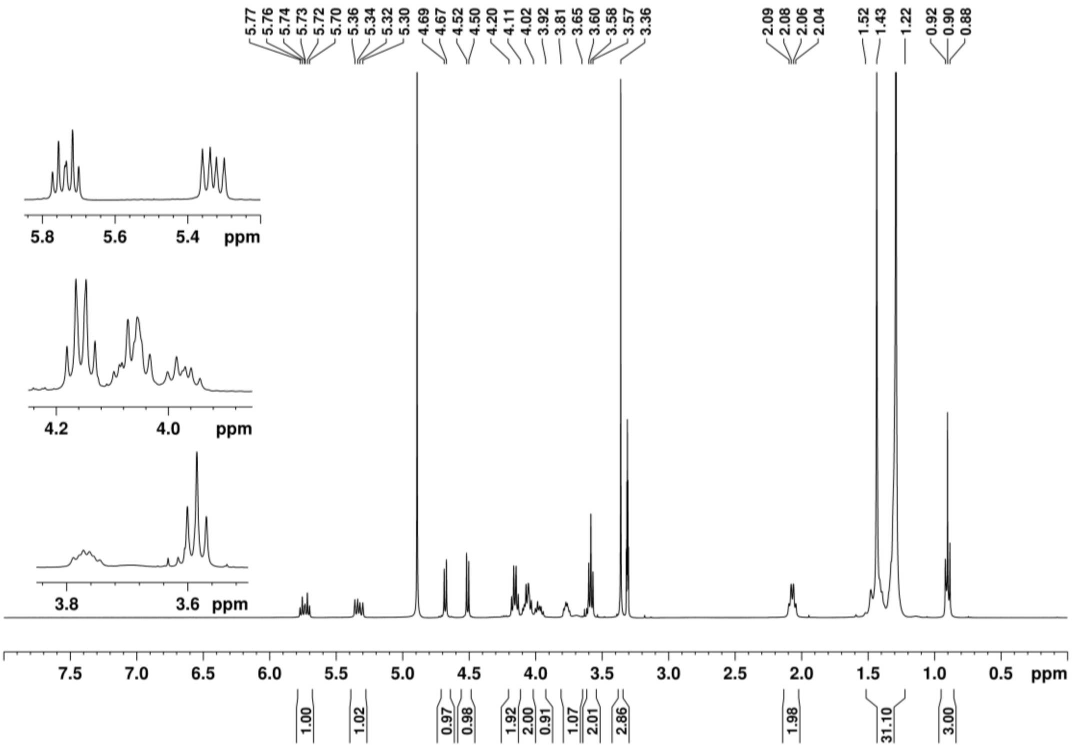

Supplementary Figure 29 ^{1}H NMR spectrum (400 MHz, CD_3OD) of compound 10.

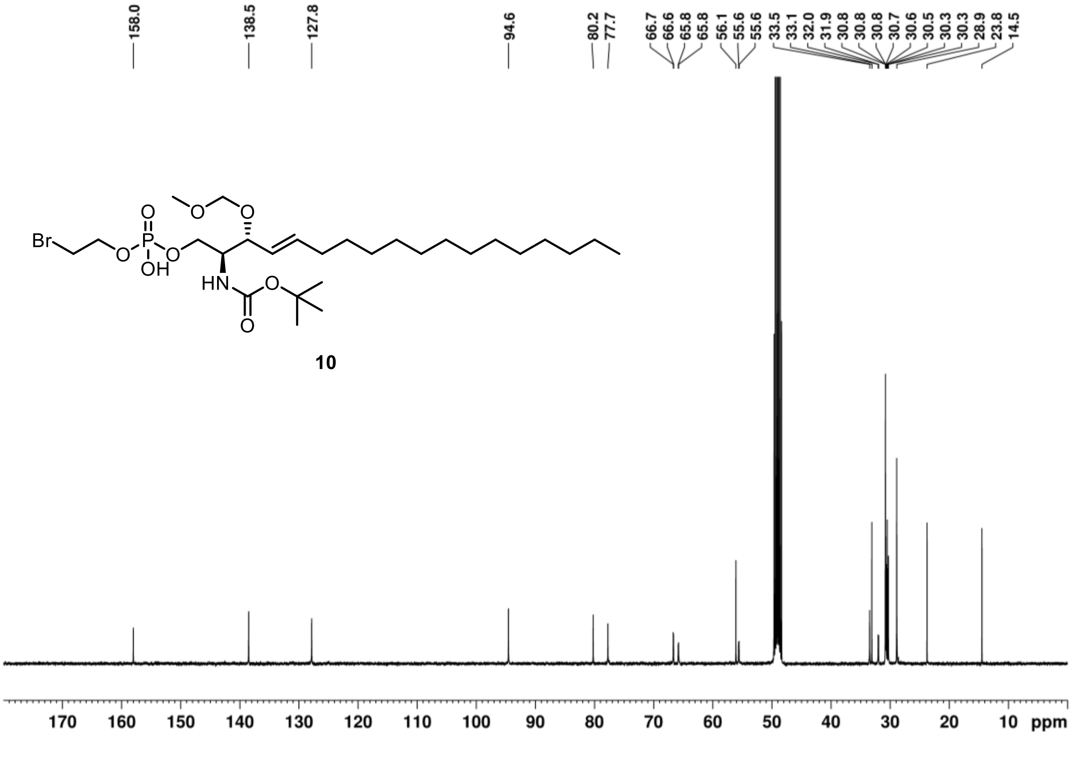

Supplementary Figure 30 ^{13}C NMR spectrum (100 MHz, CD_3OD) of compound 10.

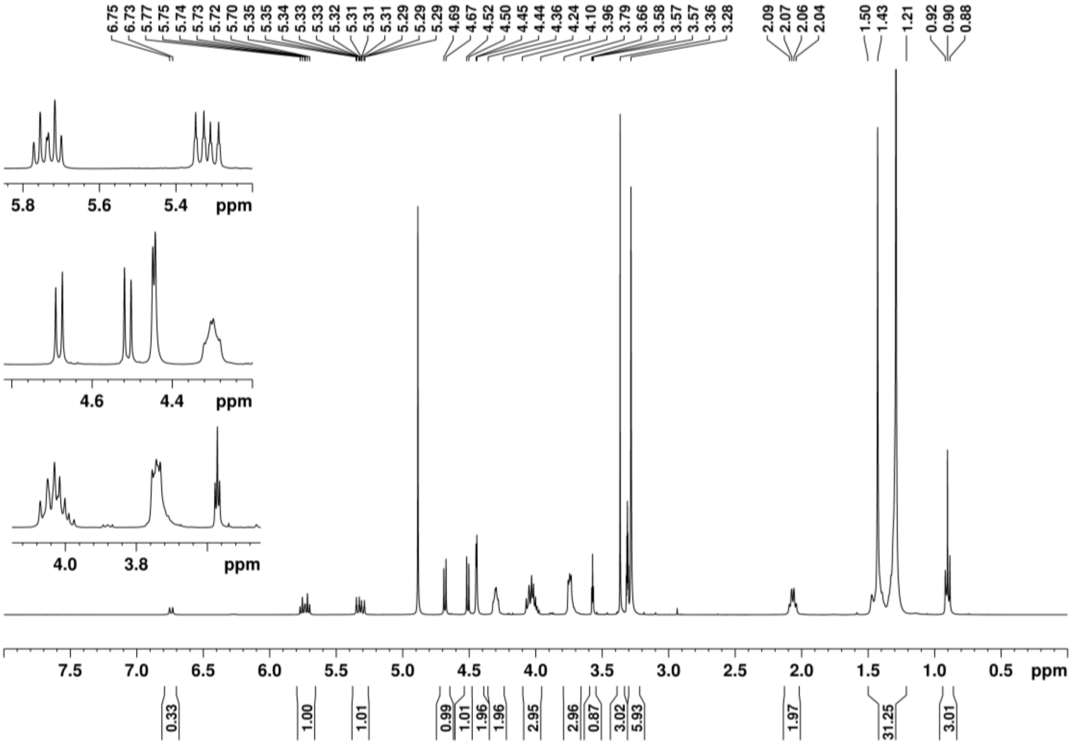

Supplementary Figure 31 ^{1}H NMR spectrum (400 MHz, CD_3OD) of compound 12.

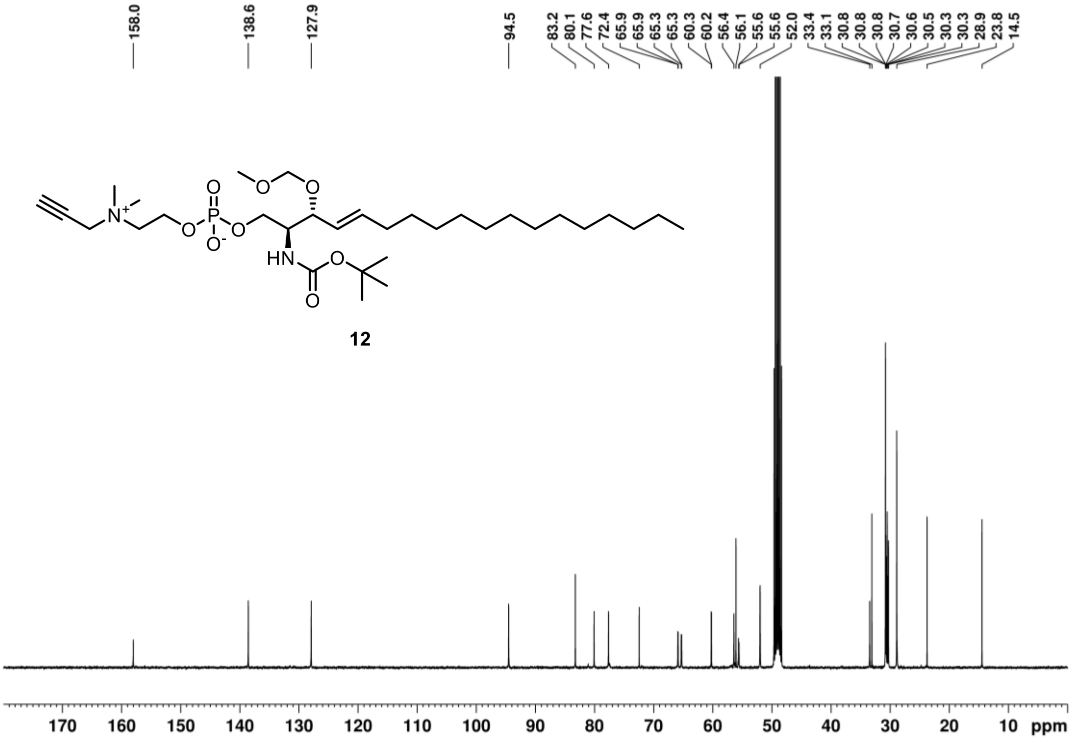

Supplementary Figure 32 ^{13}C NMR spectrum (100 MHz, CD_3OD) of compound 12.

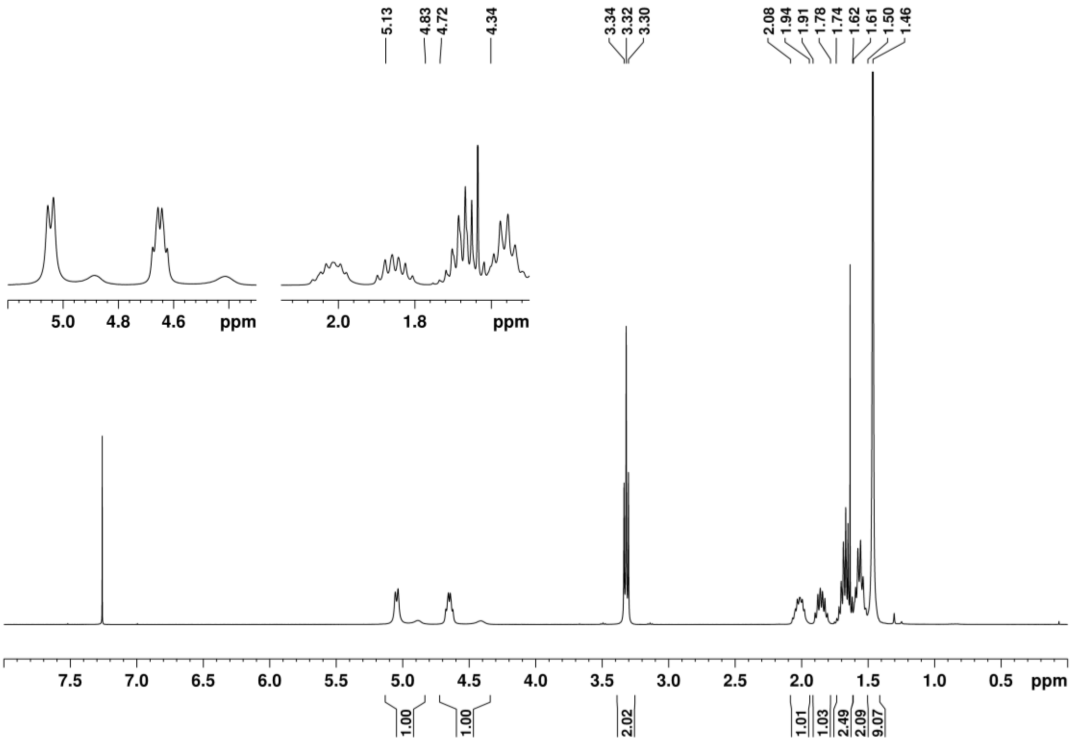

Supplementary Figure 33 ^{1}H NMR spectrum (400 MHz, $CDCl_3$) of compound 14.

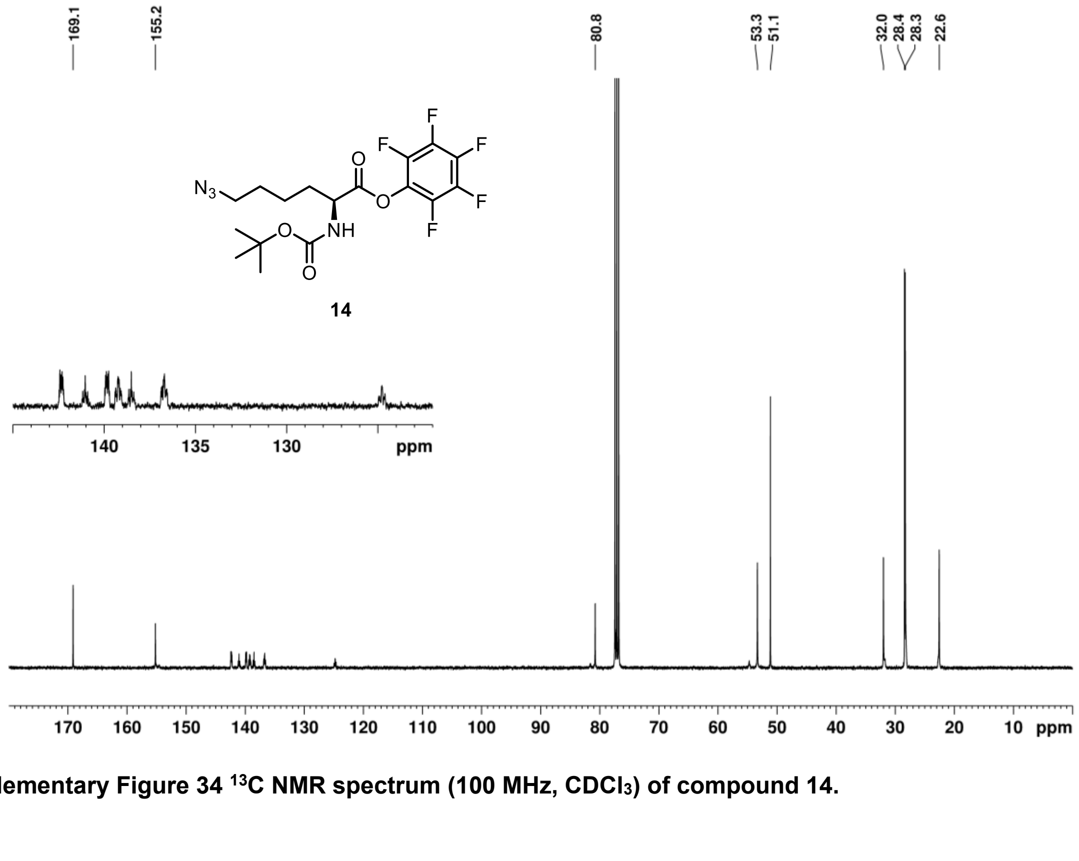

Supplementary Figure 34 ^{13}C NMR spectrum (100 MHz, $CDCl_3$) of compound 14.

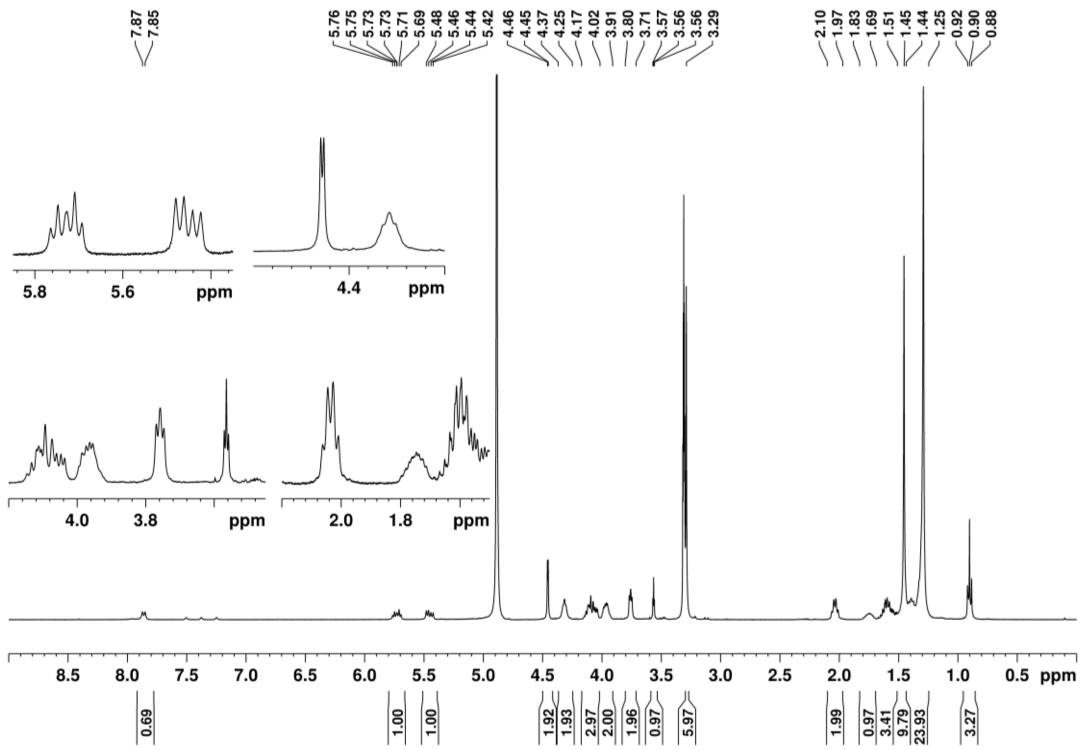

Supplementary Figure 35 ^{1}H NMR spectrum (400 MHz, CD_3OD) of compound 16.

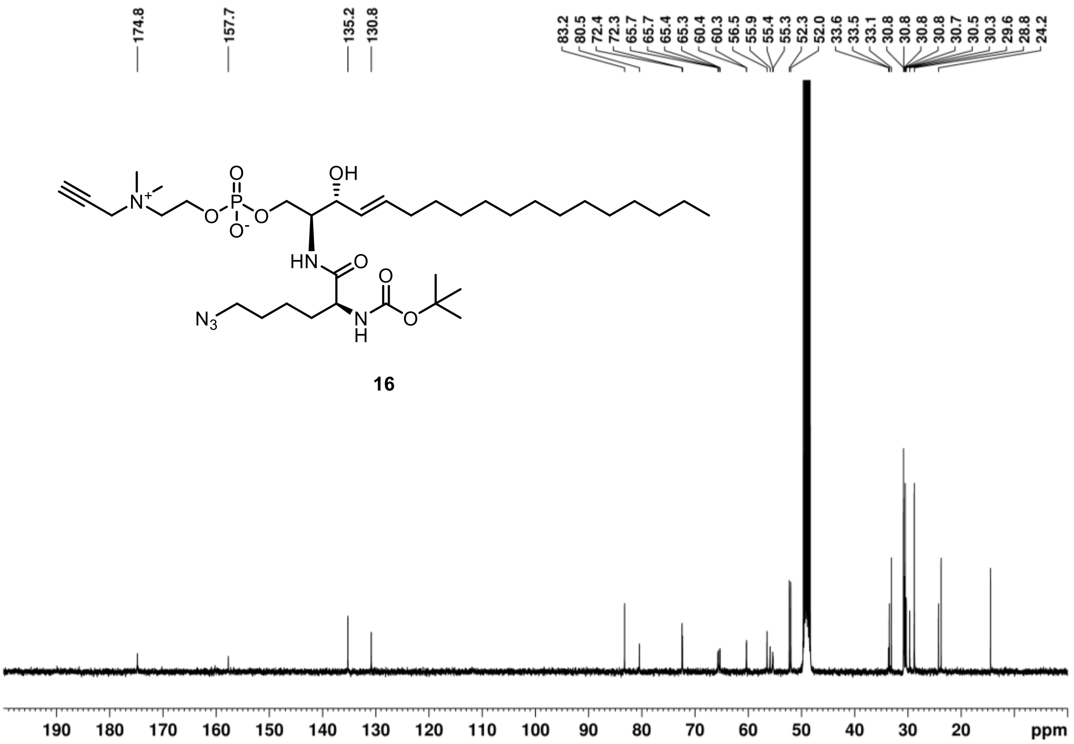

Supplementary Figure 36 ^{13}C NMR spectrum (100 MHz, CD_3OD) of compound 16.

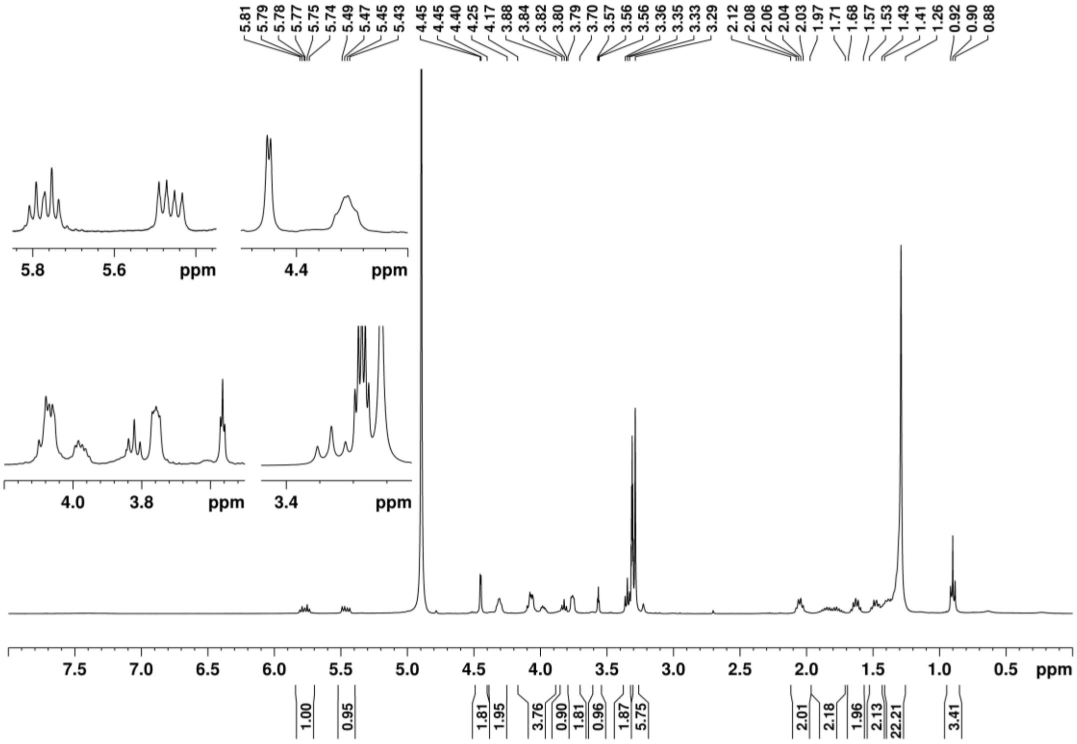

Supplementary Figure 37 ^{1}H NMR spectrum (400 MHz, CD_3OD) of target molecule TFSM2.

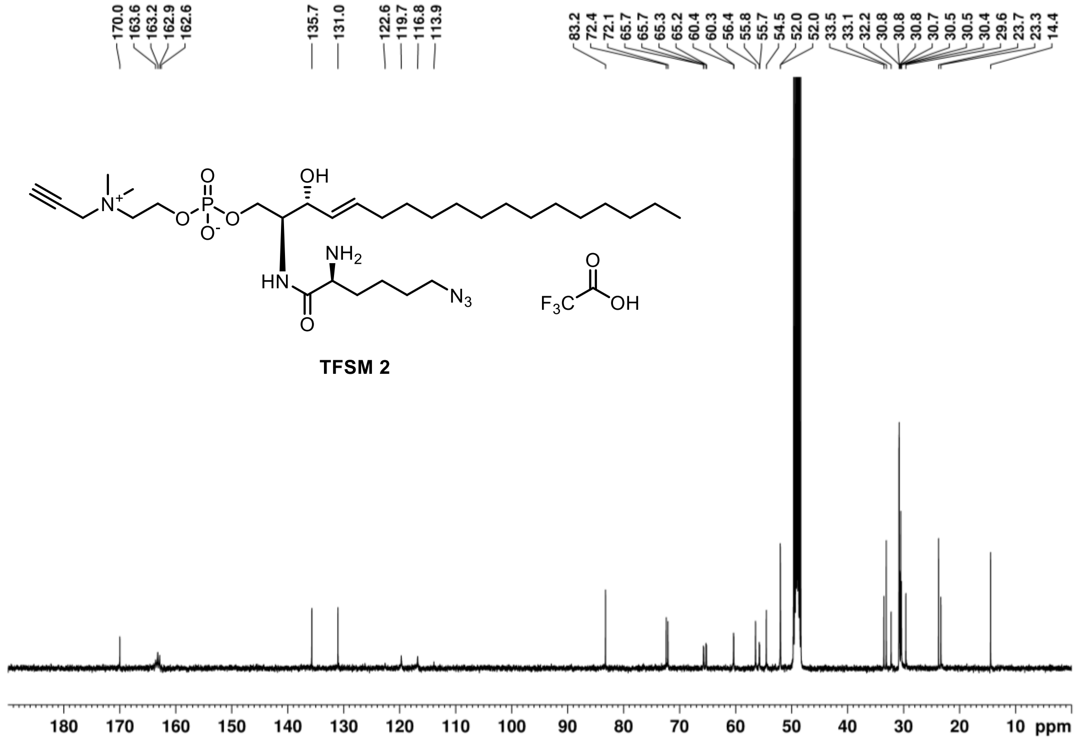

Supplementary Figure 38 ^{13}C NMR spectrum (100 MHz, CD_3OD) of target molecule TFSM2.

Supplementary Tables

Characteristics of FLIM measured infected, TFSM1-stained, HeLa cells click-labeled with BODIPY-FL-DBCO as well as BODIPY-FL-DBCO and AlexaFluor™ 546-azide. Excitation was performed at 488 nm. The decay parameters were determined by least-squares deconvolution and the quality was judged by the reduced $\chi 2$ values as well as the randomness of the weighted residuals ($\chi 2 = 0.9 – 1.4$)[8]. A multi-exponential model was used to fit all fluorescence lifetime decays.

Supplementary Table 1

figure	sample	A_1 (%)	τ_1 (ns)	A_2 (%)	τ_2 (ns)	A_3 (%)	τ_3 (ns)	$Ø_{Av., Amp.}$ (ns)
Fig. 4 e	BODIPY-FL	53.87 ± 1.96	4.72 ± 0.03	46.13 ± 1.96	1.87 ± 0.08	---	---	3.47 ± 0.20
	BODIPY-FL +AF546	16.00 ± 1.78	4.72 (fixed)	44.80 ± 1.53	2.64 ± 0.07	39.20 ± 2.92	0.94 ± 0.06	2.31 ± 0.11
Supp. Fig. 3 b	total	56.95 ± 0.01	5.17 ± 0.01	43.05 ± 0.01	1.81 ± 0.04	---	---	3.73 ± 0.02
	host membranes	38.12 ± 0.01	4.64 ± 0.04	61.88 ± 0.01	1.20 ± 0.05	---	---	2.53 ± 0.03
	inclusion 1	56.90 ± 0.02	5.21 ± 0.07	43.10 ± 0.02	1.77 ± 0.10	---	---	3.73 ± 0.02
	inclusion 2	55.9 ± 0.02	5.12 ± 0.03	44.10 ± 0.02	1.77 ± 0.09	---	---	3.64 ± 0.02
Supp. Fig.3 c + d	BODIPY-FL	56.19 ± 3.56	5.25 ± 0.13	43.81 ± 3.56	1.89 ± 0.18	---	---	3.78 ± 0.25
	BODIPY-FL +AF546	53.05 ± 0.05	5.20 ± 0.06	46.95 ± 0.05	1.76 ± 0.11	---	---	3.58 ± 0.20

Supplementary References

1. Lang, J. et al. Acid ceramidase of macrophages traps herpes simplex virus in multivesicular bodies and protects from severe disease. *Nat. Commun.* **11**, 1338 (2020).
2. Pinkert, T., Furkert, D., Korte, T., Herrmann, A. & Arenz, C. Amplification of a FRET Probe by Lipid–Water Partition for the Detection of Acid Sphingomyelinase in Live Cells. *Angew. Chem. Int. Ed.* **56**, 2790-2794 (2017).
3. Yamamoto, T., Hasegawa, H., Hakogi, T. & Katsumura, S. Versatile Synthetic Method for Sphingolipids and Functionalized Sphingosine Derivatives via Olefin Cross Metathesis. *Org. Lett.* **8**, 5569-5572 (2006).
4. Yang, R., Bi, X., Li, F., Cao, Y. & Liu, C.-F. Native chemical ubiquitination using a genetically incorporated azidonorleucine. *Chem. Commun.* **50**, 7971-7974 (2014).
5. Gulbins, A. et al. Antidepressants act by inducing autophagy controlled by sphingomyelin–ceramide. *Mol. Psychiatry* **23**, 2324-2346 (2018).
6. Naser, E. et al. Characterization of the small molecule ARC39, a direct and specific inhibitor of acid sphingomyelinase in vitro. *J. Lipid Res.* **61**, 896-910 (2020).
