# Supplementary material for: Trifunctional sphingomyelin derivatives enable nanoscale resolution of sphingomyelin turnover in physiological and infection processes via expansion microscopy
Source: Nat Commun. 2024 Aug 28;15:7456. doi: 10.1038/s41467-024-51874-w (PMC11358447; doi:10.1038/s41467-024-51874-w)
Supplement: Supplementary file 2 — Reporting Summary [file 41467_2024_51874_MOESM2_ESM.pdf]

nature portfolio

Corresponding author(s): Jürgen Seibel

Last updated by author(s): Jul 21, 2024

Reporting Summary

Nature Portfolio wishes to improve the reproducibility of the work that we publish. This form provides structure for consistency and transparency in reporting. For further information on Nature Portfolio policies, see our Editorial Policies and the Editorial Policy Checklist.

Statistics

For all statistical analyses, confirm that the following items are present in the figure legend, table legend, main text, or Methods section.

n/a	Confirmed	
☐	☒	The exact sample size (*n*) for each experimental group/condition, given as a discrete number and unit of measurement
☒	☐	A statement on whether measurements were taken from distinct samples or whether the same sample was measured repeatedly
☐	☒	The statistical test(s) used AND whether they are one- or two-sided<br>*Only common tests should be described solely by name; describe more complex techniques in the Methods section.*
☒	☐	A description of all covariates tested
☐	☒	A description of any assumptions or corrections, such as tests of normality and adjustment for multiple comparisons
☐	☒	A full description of the statistical parameters including central tendency (e.g. means) or other basic estimates (e.g. regression coefficient) AND variation (e.g. standard deviation) or associated estimates of uncertainty (e.g. confidence intervals)
☐	☒	For null hypothesis testing, the test statistic (e.g. *F*, *t*, *r*) with confidence intervals, effect sizes, degrees of freedom and *P* value noted<br>*Give P values as exact values whenever suitable.*
☒	☐	For Bayesian analysis, information on the choice of priors and Markov chain Monte Carlo settings
☒	☐	For hierarchical and complex designs, identification of the appropriate level for tests and full reporting of outcomes
☒	☐	Estimates of effect sizes (e.g. Cohen's *d*, Pearson's *r*), indicating how they were calculated

Our web collection on statistics for biologists contains articles on many of the points above.

Software and code

Policy information about availability of computer code

Data collection	Leica LAS AF Version 2.7.3.9723, Attune Cytometric Software v5.2.0, SymPhoTime64 software (PicoQuant, Version 2.8), Tecan i-control 2.0.10.0, ZEN2 blue software (Zeiss, version 3.5)
Data analysis	Flowing Software 2.5.1 (Turku Bioscience Center), Fiji (Schindelin et. al 2012, doi: 10.1038/nmeth.2019), Excel (Microsoft), GraphPad Prism Version 10.1.2., SymPhoTime64 software (PicoQuant, Version 2.8), MassHunter Quantitative Analysis software (version 10.1, Agilent Technologies)

For manuscripts utilizing custom algorithms or software that are central to the research but not yet described in published literature, software must be made available to editors and reviewers. We strongly encourage code deposition in a community repository (e.g. GitHub). See the Nature Portfolio guidelines for submitting code & software for further information.

Data

Policy information about availability of data

All manuscripts must include a data availability statement. This statement should provide the following information, where applicable:

- Accession codes, unique identifiers, or web links for publicly available datasets
- A description of any restrictions on data availability
- For clinical datasets or third party data, please ensure that the statement adheres to our policy

The authors declare that the data supporting the findings of this study are available within the paper and its Supplementary Information files. Should any raw data files be needed in another format they are available from the corresponding author upon reasonable request.

Research involving human participants, their data, or biological material

Policy information about studies with human participants or human data. See also policy information about sex, gender (identity/presentation), and sexual orientation and race, ethnicity and racism.

Reporting on sex and gender	n/a
Reporting on race, ethnicity, or other socially relevant groupings	n/a
Population characteristics	n/a
Recruitment	n/a
Ethics oversight	n/a

Note that full information on the approval of the study protocol must also be provided in the manuscript.

Field-specific reporting

Please select the one below that is the best fit for your research. If you are not sure, read the appropriate sections before making your selection.

☒ Life sciences ☐ Behavioural & social sciences ☐ Ecological, evolutionary & environmental sciences

For a reference copy of the document with all sections, see nature.com/documents/nr-reporting-summary-flat.pdf

Life sciences study design

All studies must disclose on these points even when the disclosure is negative.

Sample size	Samples size was determined empirically (e.g. based on previous studies Götz & Kunz et. al, https://doi.org/10.1038/s41467-020-19897-1 or Kunz & Rühling et. al https://doi.org/10.3389/fcimb.2021.644750)
Data exclusions	No data points were excluded from the analysis
Replication	Cell lines separtly seeded between replicates, replicates were performed on different days, partially experiments were preformed by different researchers. For number of independent experiments see figure legends.
Randomization	The study was restricted to cell lines. Each well on the well plate was considered as independent but identical experimental unit. Thus, group allocation is irrelevant to our study.
Blinding	For recording and evaluation of the FLIM measurements, Investigators were blinded. Otherwise, the Investigators were not blinded to allocation during experiments and outcome assessment.

Reporting for specific materials, systems and methods

We require information from authors about some types of materials, experimental systems and methods used in many studies. Here, indicate whether each material, system or method listed is relevant to your study. If you are not sure if a list item applies to your research, read the appropriate section before selecting a response.

Materials & experimental systems

n/a	Involved in the study
	☒ Antibodies
	☒ Eukaryotic cell lines
☒	Palaeontology and archaeology
☒	Animals and other organisms
☒	Clinical data
☒	Dual use research of concern
☒	Plants

Methods

n/a	Involved in the study
☒	ChIP-seq
	☒ Flow cytometry
☒	MRI-based neuroimaging

Antibodies

Antibodies used

anti-LAMP1 (clone H5G11, SantaCruz, Cat. No. sc-18821, Lot. #H2118, dilution 4 µg/ml); anti-GM130 (Becton Dickinson, Cat. No. 610823, Lot. 7163670, clone 35, dilution 2.5 µg/ml), anti-Prx3 antibody (OriGene, Cat. No. TA322472, dilution 1:50) anti-chlamydial HSP60 antibody (SantaCruz, Cat. No. sc-57840, Lot. #K1220, dilution 4 µg/ml) , anti-mouse CF568 (Sigma, Cat. No. SAB4600082, Lot. 16C1017, dilution 20 µg/ml), anti-mouse AlexaFluor405 (ThermoFisher, Cat. No. A48255, Lot. YA353687, dilution 20 µg/ml)

Validation

GM130: antibody raised against immunogen Rat GM130 aa. 869-982, Reactivity Rat (QC Testing), Human,Mouse,Dog (Tested in Development), Valdiated for Western blot (Routinely Tested), Immunofluorescence (Tested During Development), Immunohistochemistry, Immunoprecipitation (Not Recommended). More information can be found online: https://www.bdbiosciences.com/en-nz/products/reagents/microscopy-imaging-reagents/immunofluorescence-reagents/purified-mouse-anti-gm130.610823

LAMP1: raised against adherent spleen cells of human origin, recommended for detection of LAMP-1 of human origin by WB, IP, IF and IHC(P). More information can be found online: https://www.scbt.com/p/lamp-1-antibody-h5g11

chlamydial HSP60: raised against recombinant serovar A HSP 60 of Chlamydia trachomatis origin, recommended for detection of a C-terminal region corresponding to amino acids 401-544 of HSP 60 of C. trachomatis origin by WB, IP and IF; also recommended for detection of HSP 60 of C. psittaci, N. gonorrhoeae, with minimal cross-reactivity to B. Burgdorferi. More information can be found online: https://www.scbt.com/de/p/hsp-60-antibody-a57-b9

Prx3: Immunogen is a synthetic peptide corresponding to a region derived from 242-256 amino acids of Human peroxiredoxin 3. Applications IHC, WB. More information can be found online: https://www.biocat.com/products/TA322472-OR

Eukaryotic cell lines

Policy information about cell lines and Sex and Gender in Research

Cell line source(s)

HeLa cells (ATCC CCL-2), HeLa 229 cells (ATCC CCL-2.1), Human umbilical vein endothelial cells (HuVEC, GibcoTM, Cat. No.C01510C)

Authentication

None of the cell lines were authenticated

Mycoplasma contamination

Cell lines regularly are tested negative for Mycoplasma contamination by PCR.

Commonly misidentified lines (See ICLAC register)

No commonly misidentified cell lines were used in the study.

Plants

Seed stocks

n/a

Novel plant genotypes

n/a

Authentication

n/a

Flow Cytometry

Plots

Confirm that:

- [x] The axis labels state the marker and fluorochrome used (e.g. CD4-FITC).
- [ ] The axis scales are clearly visible. Include numbers along axes only for bottom left plot of group (a 'group' is an analysis of identical markers).
- [ ] All plots are contour plots with outliers or pseudocolor plots.
- [x] A numerical value for number of cells or percentage (with statistics) is provided.

Methodology

Sample preparation	HeLa cells were incubated with 10 µM TFSM 1, 10 µM visible range FRET probe or 1 µM BODIPYTM-FL-C12-SM (Thermo Fisher, Cat. No. D7711) in treatment medium [MCDB131 medium (GibcoTM) containing 1% (v/v) heat-inactivated FBS and GlutaMAXTM (GibcoTM)] for 2h. Then, cells were washed thrice with DPBS and treated with 100 ng/ml β-toxin for 3h. Consecutively, cells were washed thrice with DPBS (GibcoTM), detached with 300 µL/well trypsin [TrypLETM, (GibcoTM)] for 5 min at 37°C and resuspended in 300 µL 2% FBS in DPBS. For TFSM 1, samples were centrifuged (1.100xg, 5 min, 4°C) and fixed with 0.2% GA (Sigma, Cat. No. G5882) in 4% PFA in DPBS (Morphisto, Cat. No. 11762) for 30 min /RT. Cells were washed twice in 2% FBS in DPBS and then, permeabilized with 0.2% Triton X-100 (Roth, Cat. No. 6909) in PBS. Next, samples were washed twice with 2% FBS in DPBS and stained with 2 µM BODIPY-FL-DBCO (Jena Biosciences, Cat. No. CLK-040-05) in 500 µL/ samples Hanks' buffered saline (HBSS, GibcoTM) for 1h at 37°C. Samples were washed twice and stained with 10 µM AlexaFluorTM 546 azide (Jena Biosiences, Cat. No. CLK-1283) or 20 µM Tide Quencher 1 (TQ1) azide (Biomol, Cat.No. ABD-2188) in 500 µL per well click reaction buffer [50 µM CuSO4, 2.5 mM sodium ascorbate (Sigma, Cat.No. A4034), 250 µM Tris(3-hydroxypropyltriazolylmethyl)amine (THPTA; Sigma, Cat. No. 762342) in PBS] for 1h/37°C. Subsequently, cells were washed thrice with 2% FBS in DPBS. If not indicated otherwise, cells were centrifuged at 5.000xg/5 min/4°C between washing steps. Fixation, permeabilization, and staining were carried out in an end-over-end rotator. Then, cells were analyzed in an Attune NxT flow cytometer (Thermo Fischer) for BODIPY-FL (ex. 488 nm/em. band pass 530/30 nm).<br>For samples treated with BODIPY-FL-C12-SM and the visible range FRET probe, living cells were directly analyzed for BODIPY-FL/FITC (ex. 488 nm/em. band pass 530/30 nm), FRET (ex. 488 nm/em, band pass 695/40nm) and BODIPY-TR (ex.: 561nm/ em. band pass 695/40nm) with an Attune NxT flow cytometer.
Instrument	Attune NxT, ThermoFisher
Software	Attune Cytometric Software v5.2.0
Cell population abundance	n/a
Gating strategy	Events first were gated to FSC-A and SCC-A. The selected subpopulation then was gated to FSC-H and FSC-A, which yields a population that contains single cells. The mean fluorescence intensity of this population was used for qunatification.

- [x] Tick this box to confirm that a figure exemplifying the gating strategy is provided in the Supplementary Information.
